# Chemically bonded multi-nanolayer inorganic aerogel with a record-low thermal conductivity in a vacuum

**DOI:** 10.1093/nsr/nwad129

**Published:** 2023-05-08

**Authors:** Hongxuan Yu, Menglin Li, Yuanpeng Deng, Shubin Fu, Jingran Guo, Han Zhao, Jianing Zhang, Shixuan Dang, Pengyu Zhang, Jian Zhou, Dizhou Liu, Duola Wang, Chuanwei Zhang, Menglong Hao, Xiang Xu

**Affiliations:** Key Lab of Smart Prevention and Mitigation of Civil Engineering Disasters of the Ministry of Industry and Information Technology, and Key Lab of Structures Dynamic Behavior and Control of the Ministry of Education, Harbin Institute of Technology, Harbin 150090, China; Key Laboratory of Energy Thermal Conversion and Control of Ministry of Education, School of Energy and Environment, Southeast University, Nanjing 210096, China; Key Lab of Smart Prevention and Mitigation of Civil Engineering Disasters of the Ministry of Industry and Information Technology, and Key Lab of Structures Dynamic Behavior and Control of the Ministry of Education, Harbin Institute of Technology, Harbin 150090, China; Key Lab of Smart Prevention and Mitigation of Civil Engineering Disasters of the Ministry of Industry and Information Technology, and Key Lab of Structures Dynamic Behavior and Control of the Ministry of Education, Harbin Institute of Technology, Harbin 150090, China; Key Lab of Smart Prevention and Mitigation of Civil Engineering Disasters of the Ministry of Industry and Information Technology, and Key Lab of Structures Dynamic Behavior and Control of the Ministry of Education, Harbin Institute of Technology, Harbin 150090, China; Key Lab of Smart Prevention and Mitigation of Civil Engineering Disasters of the Ministry of Industry and Information Technology, and Key Lab of Structures Dynamic Behavior and Control of the Ministry of Education, Harbin Institute of Technology, Harbin 150090, China; Key Lab of Smart Prevention and Mitigation of Civil Engineering Disasters of the Ministry of Industry and Information Technology, and Key Lab of Structures Dynamic Behavior and Control of the Ministry of Education, Harbin Institute of Technology, Harbin 150090, China; Key Lab of Smart Prevention and Mitigation of Civil Engineering Disasters of the Ministry of Industry and Information Technology, and Key Lab of Structures Dynamic Behavior and Control of the Ministry of Education, Harbin Institute of Technology, Harbin 150090, China; Key Lab of Smart Prevention and Mitigation of Civil Engineering Disasters of the Ministry of Industry and Information Technology, and Key Lab of Structures Dynamic Behavior and Control of the Ministry of Education, Harbin Institute of Technology, Harbin 150090, China; Key Lab of Smart Prevention and Mitigation of Civil Engineering Disasters of the Ministry of Industry and Information Technology, and Key Lab of Structures Dynamic Behavior and Control of the Ministry of Education, Harbin Institute of Technology, Harbin 150090, China; Key Lab of Smart Prevention and Mitigation of Civil Engineering Disasters of the Ministry of Industry and Information Technology, and Key Lab of Structures Dynamic Behavior and Control of the Ministry of Education, Harbin Institute of Technology, Harbin 150090, China; Key Lab of Smart Prevention and Mitigation of Civil Engineering Disasters of the Ministry of Industry and Information Technology, and Key Lab of Structures Dynamic Behavior and Control of the Ministry of Education, Harbin Institute of Technology, Harbin 150090, China; Key Lab of Aerospace Bearing Technology and Equipment of the Ministry of Industry and Information Technology, Harbin Institute of Technology, Harbin 150001, China; Key Laboratory of Energy Thermal Conversion and Control of Ministry of Education, School of Energy and Environment, Southeast University, Nanjing 210096, China; Key Lab of Smart Prevention and Mitigation of Civil Engineering Disasters of the Ministry of Industry and Information Technology, and Key Lab of Structures Dynamic Behavior and Control of the Ministry of Education, Harbin Institute of Technology, Harbin 150090, China

**Keywords:** inorganic aerogels, multi-nanolayer, mechanical-thermal tradeoff, flexibility, thermal superinsulation

## Abstract

Inorganic aerogels have exhibited many superior characteristics with extensive applications, but are still plagued by a nearly century-old tradeoff between their mechanical and thermal properties. When reducing thermal conductivity by ultralow density, inorganic aerogels generally suffer from large fragility due to their brittle nature or weak joint crosslinking, while enhancing the mechanical robustness by material design and structural engineering, they easily sacrifice thermal insulation and stability. Here, we report a chemically bonded multi-nanolayer design and synthesis of a graphene/amorphous boron nitride aerogel to address this typical tradeoff to further enhance mechanical and thermal properties. Attributed to the chemically bonded interface and coupled toughening effect, our aerogels display a low density of 0.8 mg cm^−3^ with ultrahigh flexibility (elastic compressive strain up to 99% and bending strain up to 90%), and exceptional thermostability (strength degradation <3% after sharp thermal shocks), as well as the lowest thermal conductivities in a vacuum (only 1.57 mW m^−1^ K^−1^ at room temperature and 10.39 mW m^−1^ K^−1^ at 500°C) among solid materials to date. This unique combination of mechanical and thermal properties offers an attractive material system for thermal superinsulation at extreme conditions.

## Teaser

With chemically bonded multi-nanolayer design, inorganic aerogel overcomes the tradeoff between mechanical and thermal properties, realizing the combination of ultrahigh flexibility and record-low thermal conductivity in vacuum.

## INTRODUCTION

Since the first report of inorganic aerogels in 1931 [1], they have become one of the most attractive material families due to their unique properties, such as ultralight weight (< 10 mg cm^−3^) [2–5], large specific surface area (> 1000 m^2^ g^−1^) [6], high deformability (elastic compressive strain up to 95%) [7–9], excellent fire/corrosion resistance [3,10] and low thermal conductivity (∼15–26 mW m^−1^ K^−1^) [9,11], demonstrating promise in a wide variety of fields, including sensors, actuators, catalysis frameworks, electrodes, and especially thermal insulators [12–16]. However, inorganic aerogels are still plagued by their intrinsic tradeoff between the mechanical and thermal properties, which is the critical challenge for more reliable and wider applications. To be specific, when enhancing the thermal properties, such as thermal insulation by ultralow density and thermal stability by high crystallinity, inorganic aerogels generally become fragile due to their brittle nature or weak joint crosslinking [9,17]. While enhancing the mechanical robustness by material design and structural engineering, inorganic aerogels easily sacrifice thermal insulation and stability owing to the increased density, pore size or amorphous state [17,18]. To date, single enhancement in mechanical or thermal properties has been well studied in inorganic aerogels, but still lacks efficient synergistic strategies to solve this typical tradeoff.

To enhance the mechanical properties, the elasticity of inorganic aerogels can prevent structural cracks and collapse induced by input loadings. Studies so far have shown that the mechanical properties of inorganic aerogels can be considerably enhanced through elaborate structural design [4,9,16,19]. For example, the fiber reinforced method endowed the elastic deformability in zero-dimensional (0D) granular aerogels by the flexible fibrous network and the interfacial bonding between the fibers and the nanoparticles [20]. The physical twining or chemical bonding between inorganic fibers helped the one-dimensional (1D) fibrous aerogels obtain a more flexible framework to reduce the stress concentration and strain mismatch with less plastic deformation [4,16,21]. The hyperbolic-patterned macrostructure realized a negative Poisson's ratio behavior in the two-dimensional (2D) nanosheet stacking aerogels, which facilitated the bending and out-of-plane buckling of cell walls to transform complex stresses into a favorable compressive pattern (elastic deformability up to 95% strain) [22]. However, due to the intrinsic brittleness and insufficient cross-link junctions, inorganic aerogels still need a large amount of building blocks to guarantee the strength and toughness for better mechanical properties, which eventually increase the density and effective heat conduction pathway with undesirable increase of thermal conductivity. Reducing the density of inorganic aerogels will lead to a degraded structural integrity, which can induce the sliding and splitting of building blocks rather than elastic buckling, leading to severe strength degradation and plastic deformation [23].

To enhance the thermal properties, prior efforts mainly focused on decreasing the thermal conductivity with excellent insulating performance, and increasing the thermal stability with high working temperature [9,11,17]. A typical approach for achieving a low thermal conductivity is lowering the density of inorganic aerogels to reduce the solid heat conduction by electrons or phonons transmission (3 to 7 mW m^−1^ K^−1^ in a vacuum and 26 to 33 mW m^−1^ K^−1^ in air) [4,16–18,24]. However, with a low density, thermal radiation often becomes the dominant heat transfer mechanism, especially in a vacuum and at high temperature, which then must be mitigated by introducing infrared-absorbing species [16,17]. Moreover, the phonon scattering could be further enhanced by increasing the in-plane and out-of-plane thermal resistances: increase of material grain boundaries and defects for in-plane thermal resistance [21,25], and increase of interfaces between building blocks, such as van der Waals interface gaps, for out-of-plane thermal resistance [24]. To enhance thermal stability, much effort has been made to prevent the crystallization-induced pulverization behavior under rapid thermal shocks or high-temperature exposures (800 to 1400°C) [9,17]. Inorganic aerogels usually exhibited severe strength degradation and structural damage due to the fast grain growth rate and phase change. To this challenge, a typical pre-crystallization process has been employed in the fabrication process to realize high crystalline inorganics, such as graphene, hexagonal boron nitride, SiC and SiN aerogels [8,9,23,26,27]. Adding a phase stabilizer or endowing double/multi-phase state could also improve thermal stability by suppressing the critical phase change problem, such as the SiBCN, yttria-stabilized zirconia and mullite aerogels [28–30]. However, no matter what the enhancement of thermal properties, it can aggravate the brittleness or fragile cross-link junctions of inorganic aerogels, leading to degraded mechanical properties. Therefore, it represents a formidable challenge to realize an exceptional synergetic enhancement of mechanical and thermal properties in typical inorganic aerogels.

Here, we report a chemically bonded multi-nanolayer design and synthesis of a graphene/amorphous boron nitride aerogel (a-BNGA) to break the typical mechanical-thermal tradeoff of inorganic aerogels. The resulting a-BNGA with a low density of 0.8 mg cm^−3^ features an ultrahigh flexibility (elastic compressive strain up to 99% and bending strain up to 90%), and exceptional thermal stability (strength degradation <3% after sharp thermal shocks), as well as record-low thermal conductivity (only 1.57 mW m^−1^ K^−1^ at room temperature and 10.39 mW m^−1^ K^−1^ at 500°C in a vacuum), presenting an attractive material system for thermal superinsulation at extreme conditions.

## RESULTS AND DISCUSSION

### Fabrication and structure characterization of a-BNGA

**Figure 1.**
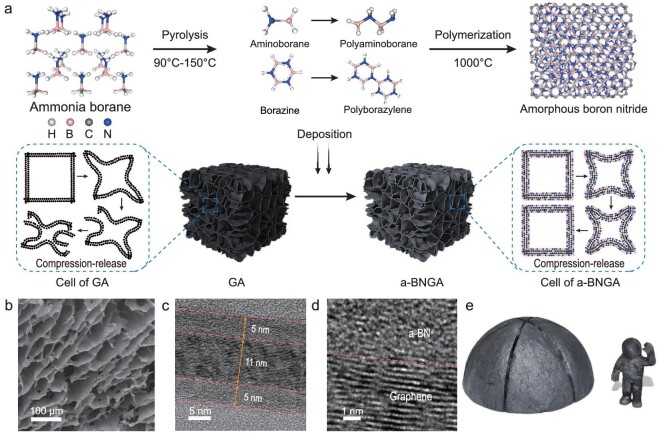
Fabrication and structure characterization of a-BNGA. (a) Illustration of the fabrication process and enhancement effect of the a-BNGA. (b) SEM image of a-BNGA framework. (c) TEM image of a-BNGA cell wall cross section with multi-nanolayer structure. (d) TEM image of a-BNGA cell wall cross section with obvious interface between a-BN and well stacked graphene. (e) Optical photograph of a-BNGA with spacesuit and lunar base shapes.

We fabricated the a-BNGA by a simple template-assisted deposition method (Fig. 1a). The graphene oxide (GO) solution was first freeze dried to obtain the graphene oxide aerogel (GOA) template. After that, the template and ammonia borane (BH_3_NH_3_, AB) powder were put in a vacuum environment with increasing temperatures for *in situ* deposition. The thermal decomposition of AB began in the temperature range of 90 to 150°C, producing hydrogen, monomeric aminoborane (BH_2_NH_2_) and borazine ((HBNH)_3_) [31,32]. Aminoborane is highly reactive and rapidly forms polyaminoborane ((NH_2_BH_2_)_n_). Then the mixture of aminoborane and borazine was employed as the precursor to freely diffuse and deposit onto the GOA framework with further polymerization via dehydrogenation. When the temperature was increased to 1000°C and maintained for 1 hour, the precursor was finally polymerized into amorphous boron nitride (a-BN) and coated onto the thermal-reduced GOA, forming the a-BNGA. Conventional neat graphene aerogel (GA) usually exhibits degraded mechanical properties due to the sliding and splitting of cell walls when reducing its density to enhance thermal insulation, while our a-BNGA may break this tradeoff by using the multi-nanolayer structural design (Fig. 1a).

**Figure 2.**
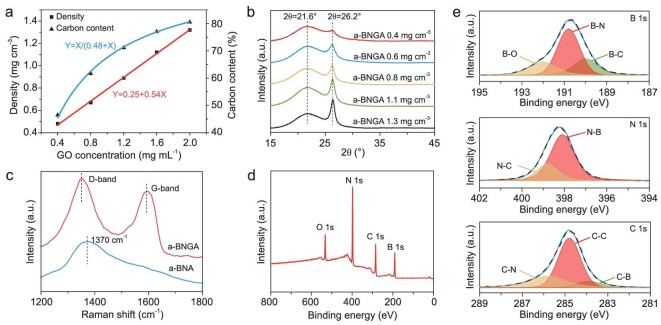
Material characterization of a-BNGA. (a) The density and carbon content of a-BNGA as a function of GO concentration. (b) XRD pattern of a-BNGA with different densities. (c) Raman spectra for a-BNGA and a-BNA. (d) XPS spectra of a-BNGA. (e) XPS spectra of B 1s, N 1s, and C 1s, respectively.

We investigated the morphology and microstructure of a-BNGA by scanning electron microscopy (SEM) and transmission electron microscopy (TEM) (Fig. 1b–d). The a-BNGA exhibits a typical three-dimensional (3D) well-assembled cellular framework with an average pore size of 10–100 μm (Fig. 1b). Interestingly, the cell walls of a-BNGA are much smoother than those of GA prepared by hydrothermal reduction, which have typical wrinkles caused by the hydrothermal process (Supplementary Fig. S1). The a-BN was uniformly deposited on both sides of the graphene, forming a multi-nanolayer structure (Fig. 1c). Since the polyaminoborane can hardly be crystallized into hexagonal boron nitride at 1000°C, the coated boron nitride nanolayers exhibit a highly disordered amorphous state (Fig. 1d) [32]. The selected area electron diffraction patterns also show typical diffuse rings (Supplementary Fig. S2), further verifying the formation of a-BN. Benefitting from our simple fabrication method and the well-assembled cellular structure, the a-BNGA can be easily scaled up with diverse shapes (Supplementary Fig. S3), such as the complex morphologies of the spacesuit and the lunar-base shell (Fig. 1e). The density of a-BNGA can be easily regulated from 0.4 to 1.3 mg cm^−3^ by tuning the contents of graphene and a-BN (Figs 2a and S4), which is comparable to the lowest values reported to date.

### Material characterization of a-BNGA

We characterized the elemental composition and chemical bond of a-BNGA using X-ray diffraction (XRD), Raman spectroscopy and X-ray photoelectron spectroscopy (XPS) (Fig. 2b–e). XRD studies reveal a typical a-BN structure with a wide diffraction peak at ∼21.6°. The characteristic peak at ∼26.2° corresponds to the reduced GO sheets with a π–π stacking pattern in the template (Fig. 2b). From the Raman spectra of a-BNGA (Fig. 2c), we only observed the typical D and G bands of reduced GO with intensity ratio (*I*_D_/*I*_G_) of 1.17. In particular, there was a blue shift of the G band from ∼1580 to 1594 cm^−1^, indicating a potential contribution of the a-BN. To further investigate this phenomenon, we thermally etched the graphene component at 600°C in air to obtain the neat a-BN aerogel (a-BNA) (Supplementary Fig. S5). A broad peak appeared at ∼1370 cm^−1^, which corresponded well to the E_2 g_ vibrational mode of a-BN [33].

XPS results verify the B, N, C and O components in the a-BNGA (Fig. 2d). From the high-resolution XPS spectra of B, N and C (Fig. 2e), the B 1 s peak can be fitted into three peaks at 190.0, 190.8 and 192.0 eV, corresponding to the B-C, B-N and B-O bonds, respectively. In the N 1s spectra, two obvious peaks at 398.1 and 398.8 eV correspond to the N-B and N-C bonds. The C 1s spectra can also be fitted into three peaks, including a main peak at 284.6 eV for C=C bonds, a wide peak at 285.8 eV for the C-N bonds, and a small peak at 283.8 eV for the C-B bonds. These results provide strong evidence for the chemical bonds between the graphene and a-BN nanolayers by forming C-B and C-N bonds. We ascribe these chemical bonds to the simultaneous dehydropolymerization of the aminoborane and borazine and the reduction of GOA with a large amount of reactive sites [34], doping B and N onto the graphene defects at the interfaces (Supplementary Figs S6 and S7). As a result, the chemically bonded interface between a-BN and graphene may enhance the structural integrity and ductility of the multi-nanolayered cell wall in a-BNGA (Supplementary Figs S8–S10).

### Mechanical properties of a-BNGA

**Figure 3.**
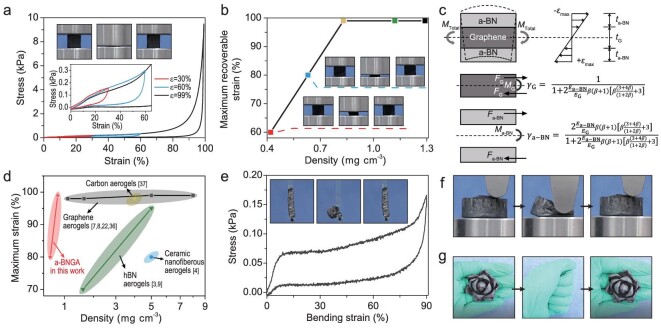
Mechanical properties of a-BNGA. (a) Uniaxial compression of a-BNGA with repeatable strain up to 99%. (Inset) The enlarged 30% and 60% strain curves and experimental snapshots of one compression cycle. The initial curves overlap well for each cycle, indicating high resilience. (b) The maximum elastic deformability of a-BNGA as a function of density. (Inset) Experimental snapshots of one compression cycle. (c) Mechanical analysis of the multi-nanolayer structure of a-BNGA. Upper half part: mechanical modeling schematic of a-BNGA with a linearly distributed stress profile along the height in the bending process. Lower half part: schematic of the equivalent concentrated force distributions applied on graphene and a-BN nanolayer, and the calculation of bending moment coefficients (*γ*) (detailed analysis as shown in Supplementary Fig. S18), where *E*_G_ and *E*_a-BN_ are the Young's modulus of the graphene and a-BN nanolayer, respectively; *t*_G_ and *t*_a-BN_ are the thickness of the graphene and a-BN nanolayer at single sides, respectively; *M*_G_ and *M*_a-BN_ are the bending moments of the graphene and the a-BN undertaken; *γ*_G_ and *γ*_a-BN_ (the ratios of *M*_G_ and *M*_a-BN_ to *M*_Total_) are the partitioned coefficients of bending moments, respectively; *β* is defined as the ratio of *t*_a-BN_ to *t*_G_. (d) The maximum strain and density of the a-BNGA compared with conventional inorganic aerogels. (e) Two-point bending of a-BNGA with repeatable strain up to 90%. (Inset) Experimental snapshots of one bending cycle. (f) Experimental snapshots of one eccentric compression cycle. (g) Experimental snapshots of an a-BNGA flower during the fold and unfold process in a human hand.

The mechanical properties of a-BNGA were first investigated by a uniaxial quasi-static compression test. Our a-BNGA with a density of ∼0.8 mg cm^−3^ exhibits superelasticity with compressive strain up to 99%, and completely recovers its original configuration after pressure release (Fig. 3a and Movie S1). This recoverable strain is notably higher than previously reported values for inorganic aerogels with ultralow density [4,8,9]. In addition, our a-BNGA can also maintain superelasticity over a large density range, showing 60% and 80% recoverable strain at densities of 0.4 and 0.6 mg cm^−3^; exhibiting 99% recoverable strain at densities of 0.8, 1.1 and 1.3 mg cm^−3^ (Figs 3b, S11 and Movie S1), and in even higher densities (Supplementary Fig. S12). The a-BNGA can be repeatedly compressed at 80% strain for 1000 cycles with small stress degradation (Supplementary Fig. S13 and Movie S2). The 1000th cycle loop remained nearly unchanged compared with the 500th cycle, and the height of the sample remained basically unchanged (residual strain <10%), indicating excellent fatigue-resistance (Supplementary Figs S13 and S14). This superelasticity can be attributed to the well-assembled chemically bonded multi-nanolayer structure in our a-BNGA (Supplementary Fig. S15). As a control experiment, the neat GA completely loses its elasticity, showing a near-zero recoverable strain (Supplementary Fig. S16). When depositing ∼0.3 mg cm^−3^ of a-BN, the aerogel begins to exhibit superelasticity (Supplementary Fig. S17). The chemically bonded interfaces tightly anchor the uniform a-BN jacket onto the graphene skeleton (Fig. 1b and c), which acts via a tendon-like mechanism, ensuring a synergistic deformation and load transfer in the framework. As a result, the armored framework can effectively reduce the sliding and splitting of cell walls when compressed, facilitating the elastic buckling of cell walls. In addition, benefiting from the robust chemically bonded interfaces, the a-BN nanolayer can increase the elastic stiffness of cell walls. We evaluated the contributions of a-BN and graphene nanolayers using the bending moment coefficient (*γ*) versus density (Figs 3c and S18). The *γ* exhibited a dramatic increase for a-BN but a notable drop for graphene with the decrease of aerogel density, indicating an effective bending moment transfer from graphene to a-BN. As a result, this a-BN–induced increase of the inertia moment of cell walls endows a desirable bending moment distribution, realizing a coupled toughening effect to enhance structural resilience. Moreover, when the graphene body is armored by the a-BN jacket, the original graphene-to-graphene van der Waals adhesion between cell walls becomes a-BN-to-a-BN, effectively reducing the adhesive force and resulting in better mechanical resilience and less plastic deformation (Supplementary Fig. S19) [35].

A different way to look at this structural enhancement is by plotting the Young's modulus (*E*) versus the density (*ρ*) as it scaled linearly as *E* ∼ *ρ*^1.61^ (Supplementary Fig. S20). This scaling corresponds well with those of superelastic inorganic aerogels with a density of 2–10 mg cm^−3^, verifying the structural enhancement in our a-BNGA with ultralow density. We also observed a negative Poisson's ratios behavior in the a-BNGA (Supplementary Fig. S21). The nanolayered cell walls can trigger out-of-plane buckling and well distribute the compressive stress to the whole structure without locally tensile fracture, both of which can enhance the performance metrics. We further investigated the superelasticity of a-BNGA over a wide range of temperatures (Supplementary Fig. S22). For example, when placed in liquid nitrogen (−196°C) or an ethanol flame (∼500°C), the aerogel can be freely compressed without any fracture. Together, our a-BNGA shows an unusual superelasticity with even lower density, which is comparable to the highest values of conventional inorganic aerogels reported to date (Fig. 3d) [3,4,7–9,22,36,37].

We subsequently evaluated the stretching, bending and torsional properties of a-BNGA (∼0.8 mg cm^−3^) with uniaxial quasi-static tension, two-point buckling and unilateral torsion method, respectively. The a-BNGA exhibits a ductile fracture strain up to ∼7.5% (Supplementary Fig. S23 and Movie S3). The bendability test reveals excellent flexibility with a bending strain up to 90% and a recoverable angle up to ∼180° without any external fracture failure (Figs 3e, S24 and Movie S3); The torsional property study shows the a-BNGA exhibits a torsional angle up to 90° with little morphology change (Supplementary Fig. S25 and Movie S3). In addition, the a-BNGA can withstand a large shear deformation from the eccentric compression with strain up to 80% (Fig. 3f and Movie S3). All the flexible deformability can be further demonstrated by the fold and unfold process of the human hand with little morphology damage (Fig. 3g and Movie S4). As a control experiment, the graphene framework coated with less a-BN exhibits larger plastic deformation under two-point buckling and lower fracture strain under quasi-static tension (Supplementary Fig. S26). This additional ultraflexibility of a-BNGA can also be attributed to its chemically bonded multi-nanolayer structure with enhanced structural integrity to prevent fracture at cross-link junctions and endow the compatible deformation mode.

### Thermal properties of a-BNGA

**Figure 4.**
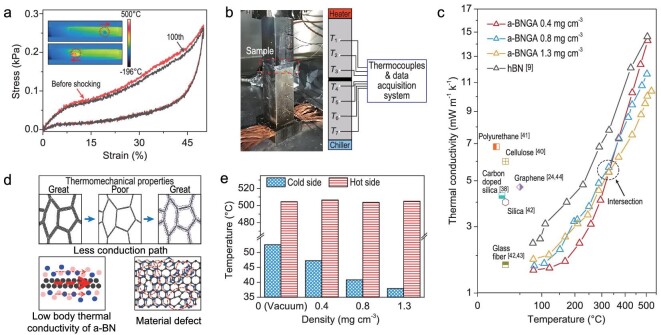
Thermostability and thermal insulation of a-BNGA. (a) The structural degradation after 100 cycles of sharp thermal shocks. (Inset) Infrared images of a-BNGA in one thermal shock cycle by using the pneumatic thermal shock testing system. (b) Setup photo (left) and schematic (right) of one-dimensional steady-state reference bar method adopted in this measurement. (c) Thermal conductivity in a vacuum of a-BNGA by our measurement and other aerogels documented from literature. (d) Upper half part: overall solid conduction path schematic. There are more paths in high-density graphene aerogel (left), good for thermomechanical properties but bad for insulation. Pure low-density graphene aerogel (center) provides fewer paths but poor thermomechanical properties. Our a-BNGA enables low-density graphene framework mechanically and thermally compatible (right). Lower half part: schematics of solid conduction path in a cell wall where a-BN nanolayer contributes very little to conduction (left) and defects within the graphene (right). (e) Temperature of cold side (*T*_4_) and hot side (*T*_3_) on the test bars in a vacuum (Supplementary Table S1); 0 (Vacuum) corresponds to no sample, thus there is only thermal radiation between hot side and cold side.

Thermal stability is a critical factor for the design and application of inorganic aerogels. We first evaluated the structural responses of a-BNGA under rapid thermal shocks by a home-designed pneumatic thermal shock testing system (Supplementary Fig. S27) [9]. The sample can be automatically moved between the cold (−196°C) and hot (500°C) ends at a frequency of 0.1 Hz (Movie S5). The mechanical strength and original morphology remained nearly unchanged (strength degradation <3% and volume shrinkage <5%) after 100 thermal shock cycles (Fig. 4a), indicating an excellent structural stability and resistance to drastic temperature changes. We also conducted a thermogravimetric (TG) analysis under air and Ar atmospheres to evaluate its thermal stability (Supplementary Fig. S28). TG curves reveal that the a-BNGA remains stable at 1000°C in Ar atmosphere with nearly no mass change. In air atmosphere, due to graphene oxidation, the beginning of primary mass loss can be observed at a temperature of 480°C. Additionally, the a-BNGA showed no strength or volume loss after one week exposure at 400°C in air atmosphere and 1000°C in vacuum condition (Supplementary Fig. S29), presenting an outstanding thermostability under high-temperature conditions.

With excellent thermal stability, the ultralight a-BNGA has great potential for thermal insulation. Moreover, graphene in a-BNGA can be used as an infrared absorber to reduce the radiative thermal transport, which is the main heat transfer mechanism for low-density aerogels at high temperatures (Supplementary Fig. S30) [17,38]. A one-dimensional steady-state reference bar method was adopted to measure the thermal conductivity of a-BNGA in high-vacuum (pressure <10^−5^ torr) [39]. The sample was sandwiched between two metal bars and a steady heat flux passed through this test column (Fig. 4b). The detailed data evaluation process is presented in Supplementary Figs S31–S33. The results show that the thermal conductivity of a-BNGA with low density at near room temperature (<100°C) in a vacuum is 1.57 mW m^−1^ K^−1^, which is, to the best of our knowledge, the lowest value for any freestanding solid material (Figs 4c, S34 and Supplementary Table S1) [9,24,38,40–44]. At near room temperature, the thermal conductivity increases with density. At high temperature (around 500°C), this trend is reversed with the higher-density a-BNGA, exhibiting the lowest thermal conductivity (10.39 mW m^−1^ K^−1^). The thermal conductivities curves of a-BNGA for three different densities intersect at around 300°C, indicating that two competing mechanisms with opposite density dependence are at play. We hypothesize that this result is due to the different effects of higher density, i.e. higher carbon loading, on heat conduction and radiation.

The solid conduction (*κ*_cond_) and radiation contributions (*κ*_rad_), which together make up the apparent thermal conductivity of a-BNGA (*κ*_total_) in a vacuum, are estimated by performing a linear fit against *T*_rad_^3^ (Supplementary Fig. S33) [9,40]. For all densities, the *κ*_cond_ of a-BNGA approaches values below the uncertainty limit of this method, indicating that *κ*_total_ is mostly contributed by radiation. We attribute this result to the scarcity of effective conduction paths. As shown in Fig. 4d, graphene conduction paths in a-BNGA are greatly reduced, resulting in ultralow solid conduction overall. Moreover, a-BN has extremely low intrinsic thermal conductivity [45] (Supplementary Fig. S35). In effect, the a-BN nanolayer in a-BNGA, which exceed 20% in volume, is mechanically crucial but thermally inactive—an ideal state for thermal insulation materials. In addition, the C-N and C-B bonds and van der Waals interface may further hinder, to some extent, phonon transport within the graphene nanolayer [46]. In fact, there is a good reason to believe that the multi-nanolayer structure can reduce the intrinsic thermal conductivity of graphene. The a-BN is coated onto the graphene nanolayer, resulting in an ‘encasing effect’ that constrains the out-of-plane phonon modes in the graphene nanolayer and induces additional phonon scattering at the a-BN–graphene interface [47]. It has been experimentally observed that this effect significantly affects the thermal transport in graphene [48].

The temperature of the test bars can reflect the thermal insulation performance of the materials in a vacuum directly as well, where we retained almost the same hot side temperature, and recorded the cold side temperature for different samples (Supplementary Fig. S31). As shown in Fig. 4e, the lower cold side temperature indicates better thermal insulation performance, which agrees well with the measured result of thermal conductivity. We also measured *κ*_total_ in the atmosphere. The a-BNGA with 0.8 mg cm^−3^ exhibits a *κ*_total_ at room temperature in atmosphere of 25.40 mW m^−1^ K^−1^ (Supplementary Fig. S36). As a control experiment, we removed the a-BNGA between the bars and tested the air effective thermal conductivity *in situ* without any change in the experimental condition. The *κ*_total_ of air obtained by this method is 25.20 mW m^−1^ K^−1^, which thoroughly agrees with the value in the literature and verifies the accuracy of our test method [49]. The difference of *κ*_total_ between a-BNGA and air at room temperature is less than 1%, which also indicates that the solid conduction in a-BNGA is effectively suppressed. In addition, we investigated the *κ*_total_ in the atmosphere at temperatures from 50 to 400°C (Supplementary Fig. S37). Our a-BNGA maintains a low *κ*_total_ of 61 mW m^−1^ K^−1^ at 400°C, exhibiting an excellent high-temperature thermal insulating performance. Since the *κ*_rad_ roughly scales with *T*_rad_^3^ at high temperatures, this result can be attributed to graphene strongly absorbing infrared radiation to effectively reduce *κ*_rad_ [17,38].

### Demonstration of thermal superinsulation for lunar base model

Lightweight, expandable and high-performance thermal insulation materials are particularly important for extraterrestrial exploration applications. For example, there is a day-night cycle of 29.5 days with large temperature difference on the lunar surface [50]. The thermal environment of the lunar base is extremely harsh, requiring superior thermal management capabilities. Although the use of insulation materials cannot heat or cool the lunar base actively, it acts as a protective layer to insulate heat flow and damp temperature fluctuations. However, transporting the large volume and weight of thermal insulation materials from Earth to extraterrestrial bases could be prohibitively expensive. Hence, benefiting from the breaking of mechanical-thermal tradeoff, our aerogel with flyweight, superelasticity (enabling a deep compress-transport-and-release cycle) and thermal superinsulation becomes highly attractive.

**Figure 5.**
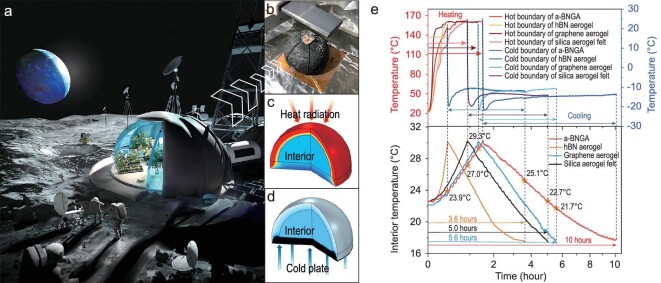
Demonstration of thermal insulation for lunar base model. (a) A schematic of the lunar base. a-BNGA serves as an outer thermal shield for a lunar base. (b) Optical photograph of the experimental set-up of lunar base model in high-vacuum. (c) Schematic of temperature field simulation and hot boundary in the heating process. At this time, the top of the base is heated as the hot boundary from about 22.5 to 160°C. (d) Schematic of temperature field simulation and cold boundary in the cooling process. The bottom of the base is then cooled as a cold boundary from about 25 to −14°C. (e) Transient temperature curves of the boundaries (upper) and interior environment (lower). The interior is heated at the beginning, after reaching 30°C, and then cooled to 17.5°C. The boundary conditions of a-BNGA, hBN aerogel, GA and silica aerogel felt used in the model are similar to ensure the comparability of demonstrations.

We designed a lunar base model working in high-vacuum to showcase the thermal insulation capabilities of a-BNGA in such applications (Fig. 5a). The hemispherical solid acts as the interior environment and is wrapped with a-BNGA on the entire surface (top hemisphere and bottom circular plane), thus simulating a lunar base shape (Fig. 5b; the detailed process is presented in Supplementary Figs S38 and S39). The a-BNGA acts as the outermost layer of the base to suppress heat exchange (through thermal radiation and conduction) with the outside environment, and the interior environment with heat capacity can store a certain amount of heat and cold. Here, we realized a simplified thermal environment for the lunar base alternating between day and night. The heating and cooling processes are implemented by controlling the hot and cold boundary conditions, respectively (Fig. 5c and d). We set the upper limit (30°C) and lower limit (17.5°C) of the interior temperature, and defined the process of the interior being heated from the initial temperature (22.5°C) to the upper limit and then cooled to the lower limit as one cycle. The aerogel with better thermal insulation can mitigate interior temperature changes more effectively, that is, have a longer cycle.

As shown in Fig. 5e, a-BNGA was demonstrated and compared to typical hBN aerogel, GA, and silica aerogel felt with the same thickness. It is obvious that the thermal insulation property of our a-BNGA is better than those of the comparative materials. Due to the small infrared radiation absorption coefficient, the interior temperature of hBN aerogel rose rapidly during the heating process, thus leading to the shortest cycle. Although GA can strongly absorb thermal radiation, the well-assembled graphene skeleton provides more phonon transport paths which obviously increase solid conductivity. The result curves show that the cooling time of our a-BNGA is 1.97 times longer than GA. For silica aerogel felt, the large density can well insulate thermal radiation, but it also provides many more solid conduction paths. The whole cycle of a-BNGA (about 10 hours) is 2.78, 1.78 and 2 times longer than that of hBN aerogel, GA and silica aerogel felt, respectively, demonstrating a-BNGA’s superior balance in suppressing thermal radiation and solid conduction. Notably, this excellent thermal insulation performance of our aerogel is well balanced with ultralow density and exceptional flexibility, showing more advantages for deep-space applications.

## CONCLUSION

Together, we have shown that a chemically bonded multi-nanolayer design and synthesis of a-BNGA with coupled toughening effect can effectively break the typical mechanical-thermal tradeoff of inorganic aerogels. The resulting material features a unique combination of exceptional mechanical and thermal properties beyond the reach of the typical inorganic aerogels, thus defining a robust material system for thermal superinsulation at extreme conditions, such as lunar and Mars bases, satellites and spacecraft. Moreover, this kind of material and structural design may also provide opportunities for inorganic aerogels to endow unique functions and break other intrinsic tradeoffs to further enhance their electrical, magnetic and optical properties for advanced battery electrode, catalyst framework, soft robot, laser lighting and adsorption devices.

## METHODS

### Fabrication of a-BNGA

GO precursor was prepared via our previous modified Hummers method [9]. To fabricate the a-BNGA, 10 mL of as-produced GO precursor (0.4–2 mg mL^−1^) was mixed with 500 μL of ethanol. The GO hydroalcoholic solution was poured into the desired mold followed by freeze-drying for 1 day. The as-prepared GOA and ammonia borane (BH_3_NH_3_) powder (0.3 g) were heated to 1000 °C at 10 °C min^−1^ under a vacuum environment for 1 hour in a quartz tube for thermal reduction of GO and *in situ* deposition of a-BN, affording a-BNGA with ∼0.4–1.3 mg cm^−3^. During the heating process, the pyrolysis products of ammonia borane freely diffused and deposited onto the framework of GOA and further polymerized into a-BN coating on a graphene framework.

### Characterization

The mechanical properties of the a-BNGA were studied by using an Instron 3365 universal testing machine equipped with a 100 N load cell, and the load rate was 10 mm min^−1^. Fatigue resistance of a-BNGA was performed with a rate of 50 mm min^−1^ for 1000 loading-unloading cycles. The sample morphology was investigated by SEM and TEM on ZEISS, Merlin Compact and FEI Talos F200X G2 microscopes, respectively. The elemental and structural analyses were carried out by X-ray photoelectron spectroscopy (XPS), X-ray diffraction (XRD) and Raman spectroscopy with 532 (for a-BNGA) and 785 (for a-BNA) nm laser excitation on Thermo Scientific K-Alpha, Panalytical X’Pert and Renishaw inVia-Reflex, respectively. The absorption spectra in 0.5–2.5 μm were measured by a UV-vis-NIR spectrometer (Lambda 950) equipped with an integrating sphere unit (density of 0.8 mg cm^−3^ and thickness of 5 mm). The thermal shock tests were carried using a home-made pneumatic system with a hot end up to 500°C and cold end in liquid nitrogen at −196°C. The infrared images were recorded with an infrared thermal camera (Flir, A615). Thermal gravimetry (TG) analysis was performed using a simultaneous thermal analyzer (TA, SDT650) at a heating rate of 10°C min^−1^. The thermal conductivities of a-BNGA in a vacuum (pressure <10^−5^ torr) and atmosphere were measured by a home-made steady-state device (heater from ∼70 to 550°C and chiller from ∼15 to 40°C). See detailed discussion in the Supplementary Information. The weight of the a-BNGA was measured with a Sartorius Micro balance (BSA124S-CW) with precision of 0.1 mg. The apparent density was calculated using the weight of solid divided by bulk of the sample.

## Supplementary Material

nwad129_Supplemental_Files
