# Supplementary material for: Chemically bonded multi-nanolayer inorganic aerogel with a record-low thermal conductivity in a vacuum
Source: Natl Sci Rev. 2023 May 8;10(10):nwad129. doi: 10.1093/nsr/nwad129 (PMC10476891; doi:10.1093/nsr/nwad129)
Supplement: nwad129_Supplemental_Files [file nwad129_supplemental_files.zip › Supplementary data.pdf]

Chemically bonded multi-nanolayer inorganic aerogel with a record-low thermal conductivity in a vacuum

Hongxuan Yu[1,†], Menglin Li[2,†], Yuanpeng Deng[1], Shubin Fu [1], Jingran Guo[1], Han Zhao[1], Jianing Zhang[1], Shixuan Dang[1], Pengyu Zhang[1], Jian Zhou[1], Dizhou Liu[1], Duola Wang[1], Chuanwei Zhang[3,]*, Menglong Hao[2,]* and Xiang Xu[1,]*

[1]*Key Lab of Smart Prevention and Mitigation of Civil Engineering Disasters of the Ministry of Industry and Information Technology and Key Lab of Structures Dynamic Behavior and Control of the Ministry of Education, Harbin Institute of Technology, Harbin, 150090, China;*

[2]*Key Laboratory of Energy Thermal Conversion and Control of Ministry of Education, School of Energy and Environment, Southeast University, Nanjing, 210096, China;*

[3]*Key Lab of Aerospace Bearing Technology and Equipment of the Ministry of Industry and Information Technology, Harbin Institute of Technology, Harbin, 150001, China*

***Corresponding authors**.

E-mail: xuxiang@hit.edu.cn; haom@seu.edu.cn; zhchwei@hit.edu.cn

[†]Equally contributed to this work.

This file includes:

Supplementary Text
Figures S1 to S39
Table S1
Captions for Movies S1 to S5
Supplementary References (1 to 21)

Other Supplementary information for this manuscript include the following:

Movies S1 to S5

Supplementary Text

Molecular dynamics (MD) simulations of a-BNGA

The reaction of GO with a-BN precursor

To better discover the reaction mechanism of GO with aminoborane, borazine, and other precursor of a-BN, we performed reactive molecular dynamics simulation at various temperature by using ReaxFF force-field [1,2]. ReaxFF is a bond-order-based empirical interatomic potential which can account for the breakage and formation of chemical bonds without taking expensive quantum mechanics calculations. The ReaxFF parameters introduced by Pai et al [3]. and Liu et al [4]. were introduced to perform the reaction simulations. A canonical (NVT) ensemble and a Berendsen thermostat was used to control the temperature of the system. Periodic condition was applied to each boundary of simulation box [5]. A time step of 0.5 fs was used for the integration of the equations of motion to ensure the stability of the simulations. All the simulations were carried out with the Large-scale Atomic/Molecular Massively Parallel Simulator (LAMMPS) code [6]. To start with, the GO sample was generated by adding 50 epoxy, 50 hydroxyls, and 50 point defects of carbon onto a 960-atom pristine single layered graphene with the size of 5 nm × 5 nm × 0.335 nm. Furthermore, each 10 molecules of aminoborane, borazine, polyaminoborane and polyborazylene, were placed randomly in a larger simulation box containing the GO sample. The final sample was treated at the temperature of 300 ℃ and 600 ℃, respectively.

Generation of atomistic model of a-BN

For the system of a-BN, we generated the sample by using the process recently reported by Liu et al [7]. We first constructed two different rectangular and square shape basic structural units as shown in Fig. S8. Then, 100 basic structural units are randomly placed in an initial packed box with the size of 10 nm × 10 nm × 10 nm. Further, a triaxial compression simulation was performed on the initial configuration to generate the a-BN model with a density of 1.1 g cm^{-3} containing 3200 atoms (Fig. S8). The model was then equilibrated at the temperature of 300 K for 10 ps to get the final atomistic model of a-BN.

The fracture simulation of a-BNGA

To evaluate the mechanical properties of a-BNGA, we performed fracture simulation and calculated the tensile strength of the a-BNGA system comparing with defective graphene by using ReaxFF force-field. The a-BNGA sample was generate by combining two 1332-atom a-BN samples with a 3-layered defective graphene. The 1332-atom a-BN sample with the size of 5 nm × 5 nm × 1 nm was generated from the equilibrated 3200-atom a-BN sample as mentioned above. Each layer of the defective graphene was generated by adding 25 carbon point defects and 25 Stone-Wales defects onto a 960-atom pristine graphene sample with the size of 5 nm × 5 nm × 0.335 nm [8]. Furthermore, the a-BNGA sample was duplicated into a 10938-atom sample with the size of 10 nm × 5 nm × 3 nm, while the 3-layered defective graphene sample was duplicated into a 5610-atom sample with the size of 10 nm × 5 nm × 1 nm. The fracture simulations were performed along the x coordinate axis for the a-BNGA and defective graphene samples, respectively. The tensile strength is obtained by increasing the box size L_x of the system stepwise by 0.25% of the initial value L_{x0} until the fracture of the fiber. After each increase in tensile strain,

the system takes a relaxation of 5 ps and then get a statistical average of stress information for another 5 ps. The stress along the x axis is then computed with the virial theorem [9]. The fracture simulation is performed with isothermal-isochoric (NVT) ensemble.

Stability of the a-BNGA under various temperature.

We further verified the stability of a-BNGA under various temperature with MD simulations by using ReaxFF force-field. The 5469-atom a-BNGA sample with the size of 5 nm × 5 nm × 3 nm was treated under the temperature of 25 ℃, 500 ℃, and 1,000 ℃ for 1 ns, respectively.

The Young's modulus of a-BN

The Young's modulus of a-BN were evaluated by using the Voigt-Ruess-Hill formalism with Tersoff interatomic potential at a temperature of 300 K [10,11]. The 3200-atom a-BN sample with the size of 3.92 nm × 3.92 nm × 3.92 nm was used.

The energy contributed by van der Waals forces of binding process of a-BN to a-BN and graphene to graphene

To show the reduction of binding energy in the system of a-BN, we performed reactive molecular dynamics simulation by using ReaxFF force-field. For the system of a-BN, two 1332-atom samples with the size of 5 nm × 5 nm × 1 nm were used, while for the system of graphene, two bilayer samples with 960 atoms and size of 5 nm × 5 nm × 0.67 nm were used. We first performed equilibration simulations for each model at the temperature of 300 K for 10 ps, and then performed the 50 ps binding simulations.

To start with, the upper and lower layers of a-BN and graphene were placed vertically with a distance of 2 nm, respectively. Fig. S19 shows that under the temperature of 300 K, the two layers of different systems would bind together as time goes by. The total binding energy and the binding energy contributed by different interatomic interactions were shown in Fig. S19. It can be found that for the system of a-BN, the energy contributed by van der Waals forces is negligible, while for the system of graphene, the $E_{van\ der\ Waals}$ takes ~40% of the total binding energy.

Thermal conductivity of a-BN

We estimated the thermal conductivity k of a-BN sample by using equilibrium molecular dynamics (EMD) with Green-Kubo linear response method [12],

$$k = \frac{1}{3Vk_BT^2}\int_0^\infty < \boldsymbol{q}(t)\boldsymbol{q}(0) > dt \tag{1}$$

where V is the volume, k_B is the Boltzmann constant, T is the temperature, $<>$ denotes the ensemble average, and $\boldsymbol{q}$ is the heat current vector defined as,

$$\boldsymbol{q} = \sum_i \sum_{j \neq i} \boldsymbol{r}_{ij} \left(\frac{\partial U_j}{\partial r_{ji}} \cdot \boldsymbol{v}_i \right) \tag{2}$$

where U_j is the site potential, and $\boldsymbol{v}_i$ is the velocity vector of atom i. The 3200-atom a-BN sample was used to compute the heat current autocorrelation function (HCACF) for 2.5 ns with a correlation length of 25 ps at the temperature of 25 ℃. 5 set of EMD simulations were performed

with different seed for the creation of initial velocities to accurately estimate the thermal conductivity of a-BN.

Basic thermal test device, theory and method

Thermal conductivity tests in atmosphere and vacuum conditions are both performed in a custom test device based on the ASTM-5470 standard [13]. The device mainly consists of two reference bars made of standard 304 stainless steel (ss). The top of the upper reference bar generates heat and the bottom of the lower reference bar generates cooling, resulting in a one-dimensional stable heat flow approximatively. The temperature of setting position in the bars is measured by thermocouples (OMEGA, K type, armored, and calibrated to ±5 mK) and collected by dada acquisition instrument (KEITHLEY DAQ6510 Data Acquisition). The sample was sandwiched between the two bars for one-dimensional steady-state thermal conductivity test.

In the steady state, if the thermal radiation and convection around the bar to the environment are ignored, the heat flux through the bar can be directly calculated by Fourier law.

$$q = \kappa_{ss}\frac{dT}{dz} \tag{1}$$

where, κ_{ss} is the thermal conductivity of the bar. Because of the large temperature gradient of the bar, the thermal conductivity value used is temperature-dependent. We adopted the conductivity documented from literature [14]. T is the temperature of the bar, which is equal in the same cross section and linearly dependent on the coordinate z in this condition. At this point, the temperature of the upper and lower interfaces of the sample (T_{0_+}, T_{0_-}) can be easily obtained by linear fitting of temperature test points. Thus, the thermal conductivity of the sample can be calculated by Eq.2.

$$\kappa_{sample} = \frac{q \times \delta}{T_{0_+} - T_{0_-}} \tag{2}$$

where, δ is the thickness of sample, which is measured by a situ displacement meter accurate to micron fixed on the upper bar.

Refined thermal conductivity test in atmosphere

In practice, the thermal radiation and convection around the bar to the environment cannot be ignored. The heat transfer of bar and sample must match the corresponding partial differential equation of heat conduction.

$$\frac{\partial}{\partial z}\left(\kappa_{ss}A\frac{\partial T}{\partial z}\right) = q_{bar.conv.} + q_{bar.rad.} \tag{3}$$

$$\frac{\partial}{\partial z}\left(\kappa_{sample}A\frac{\partial T}{\partial z}\right) = q_{sam.conv.} + q_{sam.rad.} \tag{4}$$

where, A is the cross-sectional area of the bar. $q_{bar.conv.}$ and $q_{bar.rad.}$ are the thermal convection flux and the thermal radiation flux around the bar to environment, respectively. $q_{sam.conv.}$ and $q_{sam.rad.}$ are the thermal convection flux and the thermal radiation flux around the sample to environment, respectively. If the sample center is regarded as the origin of coordinates, corresponding thermal boundary conditions should exist at the contact interface ($z_{0_+} = +{}^{\delta}/_{2}, z_{0_-} = -{}^{\delta}/_{2}$)

between bars and the sample, respectively.

$$\kappa_{ss}A(\frac{\partial T}{\partial z})_{0_+} = \kappa_{ss}A(\frac{\partial T}{\partial z})_{0_-} + q_{sam.conv.} + q_{sam.rad.} \quad (5)$$

Due to the extreme low thermal conductivity of the sample, the thermal contact resistance in the testing process is small to be ignored compared with the thermal body resistance. Then the heat flux is continuous and equal at contact interfaces.

$$\kappa_{ss}A(\frac{\partial T}{\partial z})_{0_+} = \kappa_{sample}A(\frac{\partial T}{\partial z})_{0_+} \quad (6)$$

$$\kappa_{ss}A(\frac{\partial T}{\partial z})_{0_-} = \kappa_{sample}A(\frac{\partial T}{\partial z})_{0_-} \quad (7)$$

It is not difficult to see from the above governing equation that it is the key to solve the thermal conductivity of the sample by eliminating values of thermal convection and radiation to calculate the one-dimensional thermal conduction. Especially, the one-dimensional thermal conduction in the bar axis is even no longer the main of the total heat transfer because of the amounts of thermal convection when the thermal resistance of sample is very large. To mitigate this effect, some measures are needed to reduce thermal convection. Commercial aerogel with a thickness of 10 mm was applied around the bars and sample to accomplish this improvement.

The effects of thermal convection and radiation are considered when processing the test data in order to obtain more accurate results. We use finite element simulation to refine the heat transfer of the entire test unit. The temperature points read by the experiment are used to nonlinearly fit the temperature points simulated by the finite element. κ_{sample} as an unknown parameter is given an initial value and then iterated until fitting is optimal (the variance between the experimental values and the simulated values is minimal) (Fig. S36).

<u>Refined thermal conductivity test in vacuum</u>

Here, $q_{bar.conv.}$ and $q_{sam.conv.}$ do not exist because there is no convection in vacuum. The thermal conductivity of a-BNGA drops to a very low level, generating a large thermal resistance. At this time, the q of the upper reference bar decreases in the proportion of the total heat flow, or even no longer dominates, because of the large radiation loss. More accurate results can be obtained by evaluating the heat transfer of the lower bar using the method of the test in atmosphere. The fitting curve and sensitivity analysis on one thermal conductivity value are shown in the Fig. S32.

<u>Estimation of solid and radiation contribution to the total thermal conductivity</u>

In vacuum, the total thermal conductivity we tested includes contributions of the pure solid conduction κ_{cond} and the radiation κ_{rad}. Since κ_{rad} and κ_{cond} have very different temperature dependences, their contributions can be evaluated separately [15]. Assuming that the properties (absorption and scattering coefficients) of the radiant material inside the a-BNGA is temperature independent, κ_{rad} is proportional to T_{rad}^3 (Eq.8). Since κ_{cond} is only a weak function of temperature, it can be regarded as independent of T_{rad}^3. Therefore, based on the total thermal conductivity test results, we can separate the κ_{cond} and κ_{rad} through simple linear fitting (Fig. S33). At this point, the intercept of the fitting curve can be approximated as κ_{cond}.

$$T_{rad}^3 = \frac{1}{4} \times (T_{hot}^2 + T_{cold}^2) \times (T_{hot}+T_{cold}) \quad (8)$$

It is not difficult to see from the fitting curves of three a-BNGA with different densities that the intercepts are all extremely small. Especially for the first two lower-density a-BNGA, the intercept of the fitting curve is negative. Negative κ_{cond} is obviously impossible, which we attribute to the simplistic use of linear fitting to separate κ_{cond} and κ_{rad}. After all, the factors assumed by this method have certain error on the fitting. However, the fitting results can confirm that the κ_{cond} of a-BNGA is tiny, even negligible compared to κ_{rad}. Moreover, with the decrease of a-BNGA density, the κ_{cond} decreases further.

Temperature records of cold side (T_4) and hot side (T_3) on the test bars in vacuum

The temperature of the test bars can reflect the thermal insulation of the materials in vacuum directly as well (Fig. S31). We only changed samples in the test and controlled the bottom temperature of the upper test bar (hot side, T_3) to be around 500 ℃ (floating less than 5 ℃), and ensured that any other experimental conditions are fixed. At this time, the better the thermal insulation of the sample, the lower the temperature at the top of the lower test bar (cold side, T_4). The result shows that, the cold side temperature was increased to 52.59 ℃ by pure radiation when there is no sample sandwiched in test setup (a vacuum between the bars). The cold side temperature will gradually decrease as a-BNGA with higher densities are applied in sequence, which accords with the test result of thermal conductivity perfectly.

Demonstration of thermal insulation for lunar base model by aerogels

The entire unit is demonstrated in vacuum (Figs S38 and S39). The interior in the lunar base model is suspended using three copper wires with a diameter of 0.05 mm at a distance of 5 mm from the ground to ensure that aerogel of the corresponding thickness can be filled. Since the indoor environment of the lunar base is definitely adapted to the atmospheric environment of the living organisms, it has a certain heat capacity. Thus, we chose Q235B steel as interior material for heat and cold storage (specific heat capacity: 470 J kg^{-1} K at 20 ℃, thermal conductivity: 53-49 W m^{-1} K^{-1} at 0-100 ℃, documented from literature) [16]. Thus, the whole base has certain passive thermal management ability, that is, it can have certain self-adaptability under extremely hot or cold conditions.

The thermal environment on the lunar surface is complex. During lunar day, the lunar surface is directly exposed to thermal radiation from the sun, and the highest temperature can reach 127 ℃; During lunar night, a large amount of heat will flow into space through thermal radiation due to the absence of atmosphere protection, and the surface temperature can reach as low as -180 ℃ [17]. The heat source suspended above the base provides hot boundary condition for the upper surface through thermal radiation, thereby realizing the controllable heating process. The cold plate under the base provides cold boundary condition for the lower surface through contact thermal conduction, thereby realizing the controllable cooling process.

We use thermocouples to measure temperature points: hot boundary, indoor, and cold boundary. Heating and cooling consist of a cycle:

Heating process

The silicon nitride heating sheet suspended above the base model is energized, generating thermal source and transferring heat to the outer surface of the aerogel *via* thermal radiation. We control the heating sheet by adjusting the power supply to ensure that the thermal boundary temperatures are equal when using different aerogel examples. The heat flow is transferred to the interior through the aerogel layer. Here, the starting temperature (~22 to 30 ℃) of the interior is considered as the heating process. Although the bottom aerogel is in contact with the ground, due to the small temperature difference between the interior and the ground, the heat transferred by the interior to the ground through the bottom aerogel is negligible. At this point, the cold boundary can be considered adiabatic. As can be seen from above process, the lower the total thermal conductivity of the aerogel and the better the thermal insulation, the slower the interior temperature rises (the longer the heating process).

Cooling process

The interior comes into contact with the cold plate by aerogel. Coolant flowing through the cold plate at around -40 ℃ cools the surface. Here, an indoor temperature of 30 to 17.5 ℃ is considered as a cooling cycle. The aerogel on the upper side of the interior only transfers very little thermal flow through radiation to the environment in vacuum. At this point, the hot boundary can be considered adiabatic. It is not difficult to see that the lower the thermal conductivity of the aerogel, the slower the interior temperature drops (the longer the cooling process).

Demonstration feasibility analysis

We start from the partial differential equation of heat conduction to analyze the unsteady thermal conduction in this demonstration, in order to verify the correlation between good thermal insulation of aerogel and long cycle of the base model. Here, we take the interior as the research object and we consider the thermal resistance generated by the aerogel layer covering the interior as the heat transfer resistance on the surface of the interior. From the above boundary conditions, we can see that the thermal conduct resistance inside the interior is much smaller than the heat transfer resistance on the surface of the interior. Here the Centralized Parametric Method is applicable [18].

$$\frac{dt}{d\tau} = -\frac{Ah(t - t_\infty)}{\rho c V} \tag{9}$$

$$\leftrightarrow \frac{t - t_\infty}{t_0 - t_\infty} = exp\left(-\frac{hA}{\rho c V}\tau\right) = exp\left(-\frac{\tau}{\tau_c}\right) \tag{10}$$

$$\tau_c = \frac{\rho c V}{hA} \tag{11}$$

where, t and t_0 are the real-time temperature and initial temperature of the interior, respectively. t_∞ is the environment temperature, which is the temperature of hot boundary in heating process or cold boundary in cooling process here h is the total external surface heat transfer coefficient of interior. ρ, c, V and A are the density, heat capacity, volume and surface area of interior, respectively. τ is the time. Here, we introduce the time constant τ_c. The larger the τ_c, the slower the temperature changes. Therefore, we can verify that the better thermal insulation of the aero-

gel, the slower the interior temperature changes (the longer the cycle).

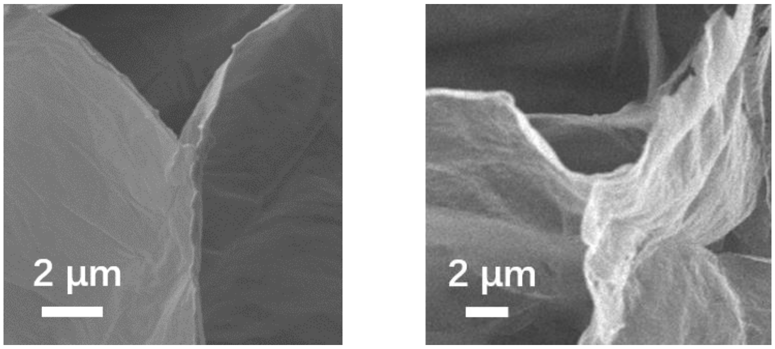

Figure S1. SEM images of "Y-shaped" joint of cell walls in a-BNGA (left) and graphene aerogel (right).

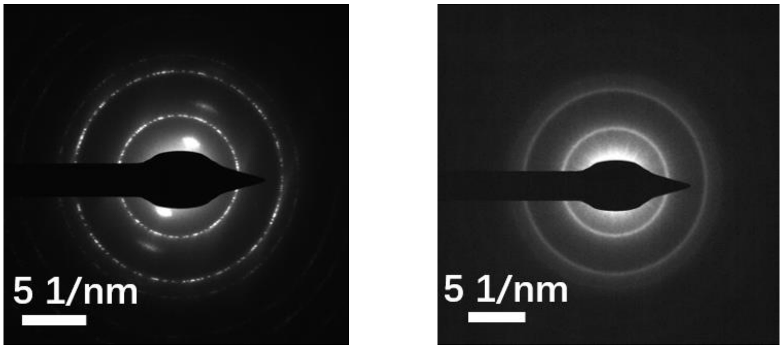

Figure S2. SAED of a-BNGA (left) and a-BN (right).

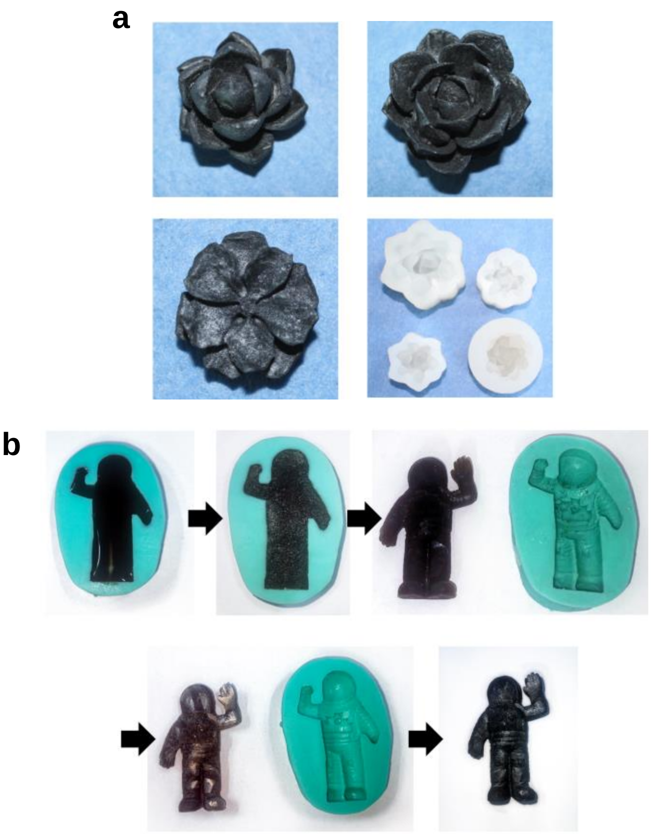

Figure S3. (a) Optical photographs of a-BNGA with complex flower shapes and the silicone molds used in the preparation process. (b) The fabrication process of a-BNGA with complex shapes.

For the a-BNGA with spacesuit shape, we used silicone molds. For the silicone molds with specific shapes, the designed structure is firstly printed using a 3D printer with a commercial resin. Then an inverse silicone mold is prepared using the cured resin structures. Note that the silicone mold can be repeatedly used. The as-prepared GO hydroalcoholic solution is filled into the desired mold method, the formed structures can be easily taken out of the mold after being completely frozen.

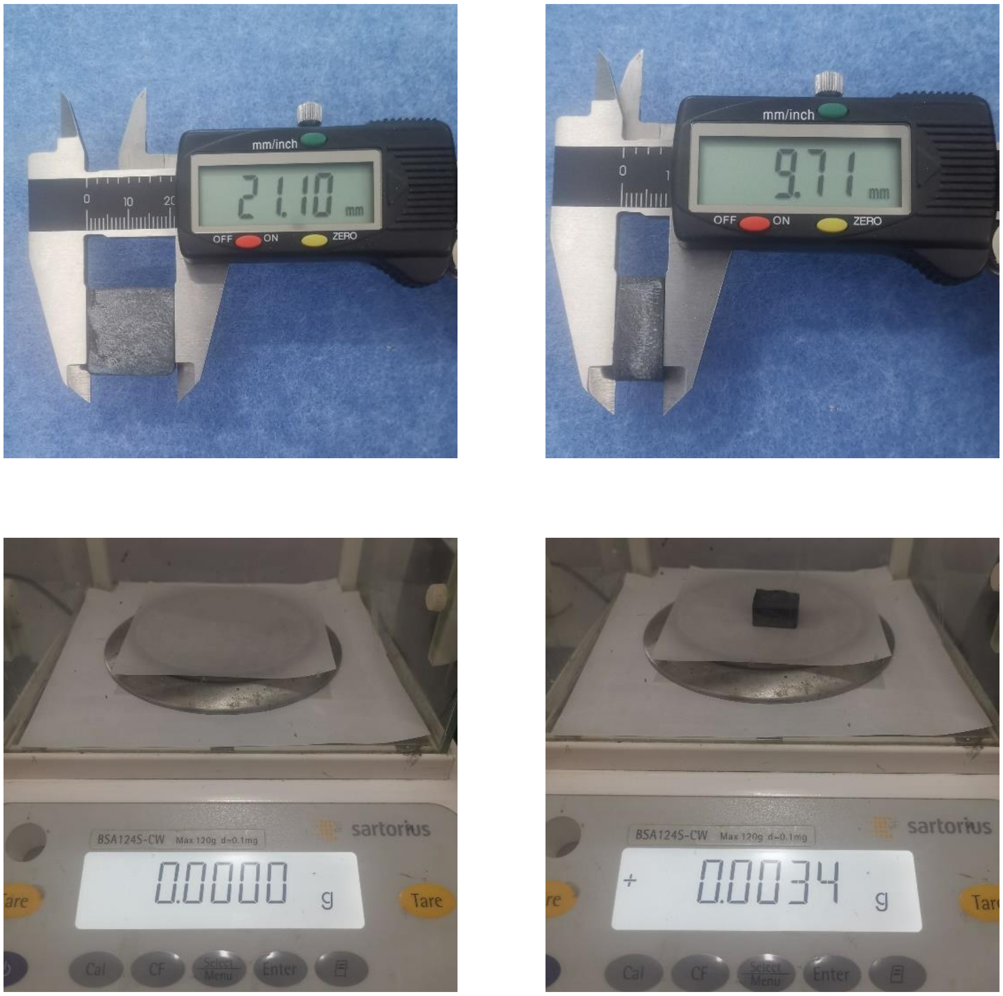

Figure S4. Density measurement of a-BNGA sample in ambient conditions. The weight measurement process of the a-BNGA (4.32 cm^3) with a mass of 3.4 mg corresponds to a density of ~0.8 mg cm^{-3}. We selected GO concentration as a variable and prepared a-BNGA with different densities.

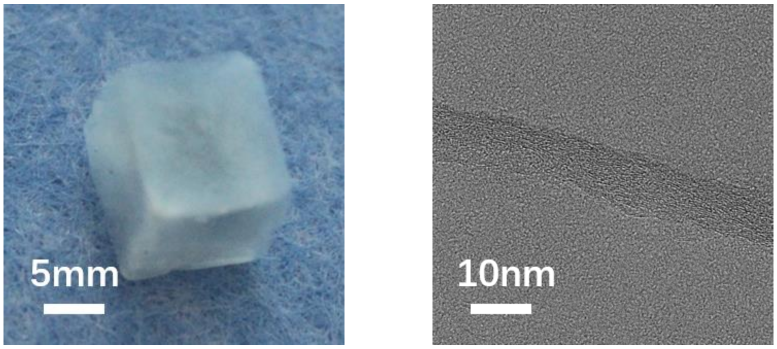

Figure S5. Optical photograph and TEM image of a-BNA.

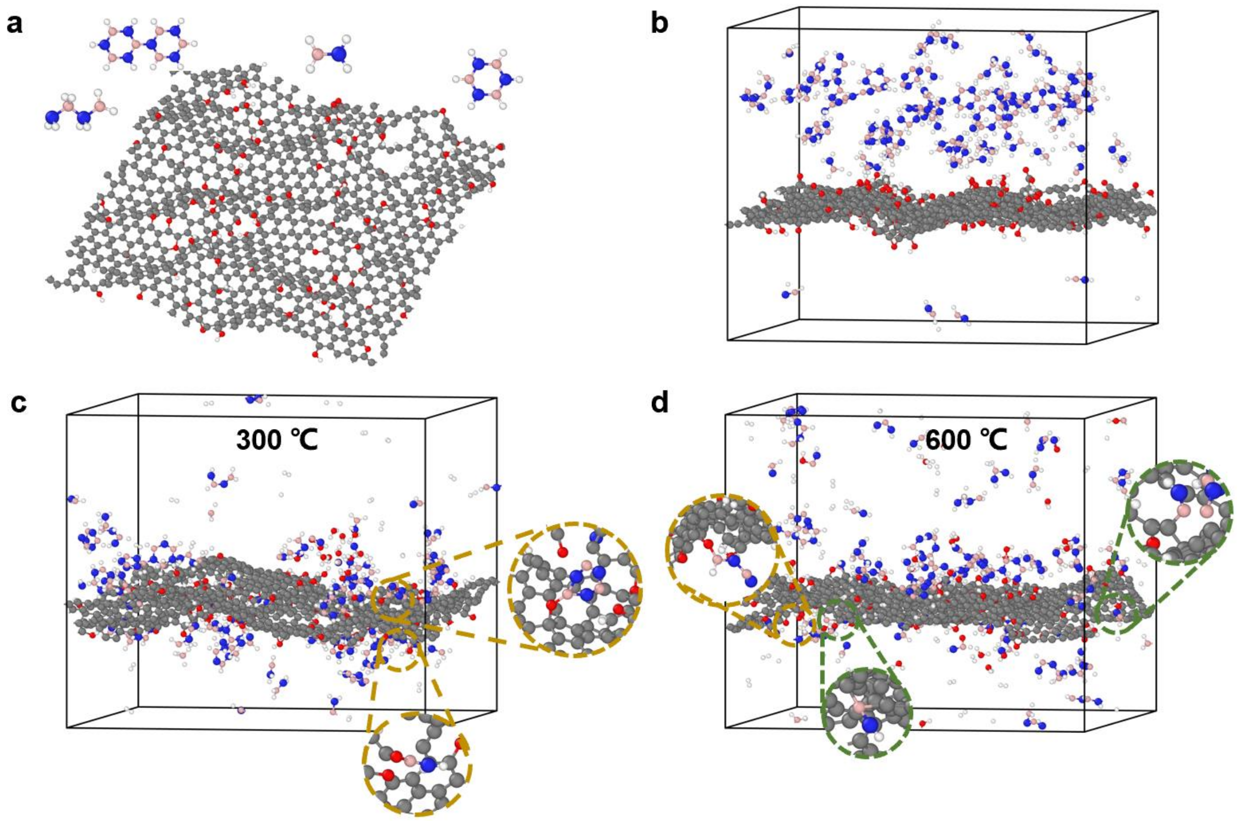

Figure S6. (a) Schematic diagram of the generation of GO model under the atmosphere of aminoborane, borazine, polyaminoborane and polyborazylene. (b) Snapshot of the initial system. Snapshot of the system after 5 ns of MD simulation at (c) 300 ℃ and (d) 600 ℃, respectively.

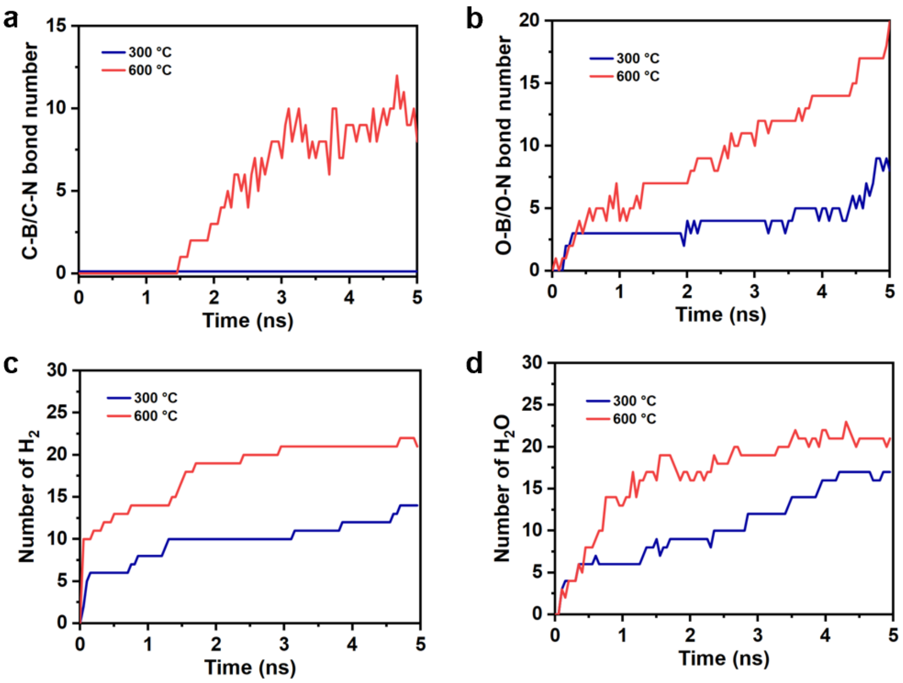

Figure S7. (a) Sum of C-B/C-N bond number, (b) sum of O-B/O-N bond number, (c) number of H_2 molecule, and (d) number of H_2O molecule as function of simulation time at the temperature of 300 ℃ and 600 ℃.

Based on the simulation results, we consider the precursor of a-BN would react with GO through two basic mechanisms. First, the precursor would form covalent bond with GO through the oxygen atom of hydroxyl and epoxy group at 300 ℃ (Fig. S6), while the increase in O-B/O-N bond number at 600 ℃ (Fig. S7b) indicates the increase in temperature would accelerate the reaction rate. Second, direct C-B/C-N covalent bonding would form at the point defect of GO at temperature of 600 ℃, while at 300 ℃, no bonding between carbon and boron/nitrogen was observed. Besides, the increase of temperature would arise the reaction rate of the dehydrogenation of precursors and the reduction of GO (Fig. S7c and d), thereby promote the growth of a-BN on the surface of graphene. It should be mentioned that due to the limited simulation time in MD simulation, the result can only provide qualitative mechanism of the reaction between GO and other precursors. A slightly higher reaction temperature was used to accelerate the reaction in the MD simulations.

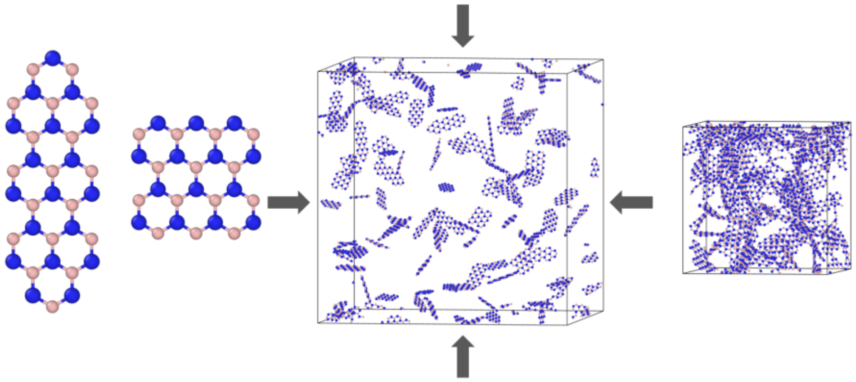

Figure S8. Snapshots of generation of atomistic model of a-BN.

Basic structural units with rectangular and square shape to generate the initial atomic configuration. Final atomistic model of a-BN with a density of 1.10 g cm^{-3} matches our estimated actual a-BN bulk density.

The cell walls of a-BNGA can be simplified as a mechanical model of a sandwich slab. According to the cell walls are regarded as the basic unit of aerogel, we can estimate the a-BN bulk density from the TEM image of a-BNGA cell wall cross section (Fig. 1c), given the known density and carbon content of a-BNGA sample (Fig. 2a):

$$\frac{\rho_G \times t_G}{\rho_{a-BN} \times 2t_{a-BN}} = \frac{\rho_{GA}}{\rho_{a-BNA}}$$

where t_G and $t_{a\text{-}BN}$ are the thickness of the graphene framework and a-BN nanolayer at single sides, respectively (nm); ρ_G and ρ_{GA} are the graphite bulk density and graphene volumetric content; $\rho_{a\text{-}BN}$, and $\rho_{a\text{-}BNA}$ are the a-BN bulk density and a-BN volumetric content. The value of ρ_G is 2.2 g cm^{-3}, and the sample density used in the TEM test was ~0.8 mg cm^{-3}. The density of a-BN bulk is estimated to 1.10 g cm^{-3}.

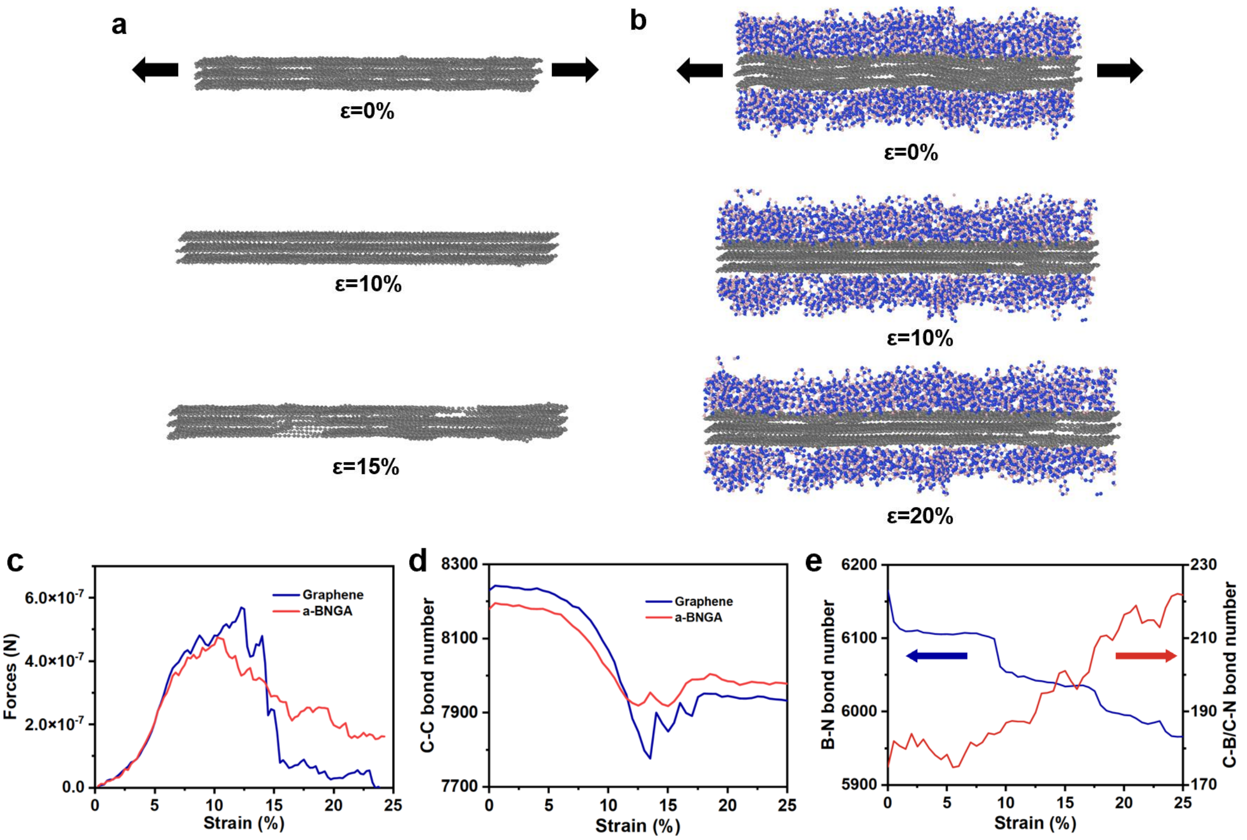

Figure S9. Snapshots of (a) defective 3-layer graphene under initial state, 10% strain and 15% strain, (b) a-BNGA system under initial state, 10% strain and 20% strain. (c) Forces as function of tensile strain in the system of graphene and a-BNGA. (d) Number of C-C bond as function of tensile strain in the system of graphene and a-BNGA. (e) Number of B-N bond and the sum of C-B/C-N bonds as function of tensile strain in the system of a-BNGA.

As shown in Fig. S9, the defective graphene sample would show brittle fracture at 0.1475 strain, while the maximum of tensile force appears at 0.1225 strain with force of 5.69×10^{-7} N. The a-BN layer would significantly improve the ductility of the a-BNGA sample and exhibited a ductile failure at over 0.2 strain. We further analyzed the number of covalence bond during the fracture simulation. The C-B/C-N bond would increase with the strain applied on the sample, indicating enhance the structural integrity of the chemically bonded interface between a-BN and graphene. At the interface the a-BN would form covalence bond with graphene at the breakage of graphene, which thereby increase the ductility of a-BNGA.

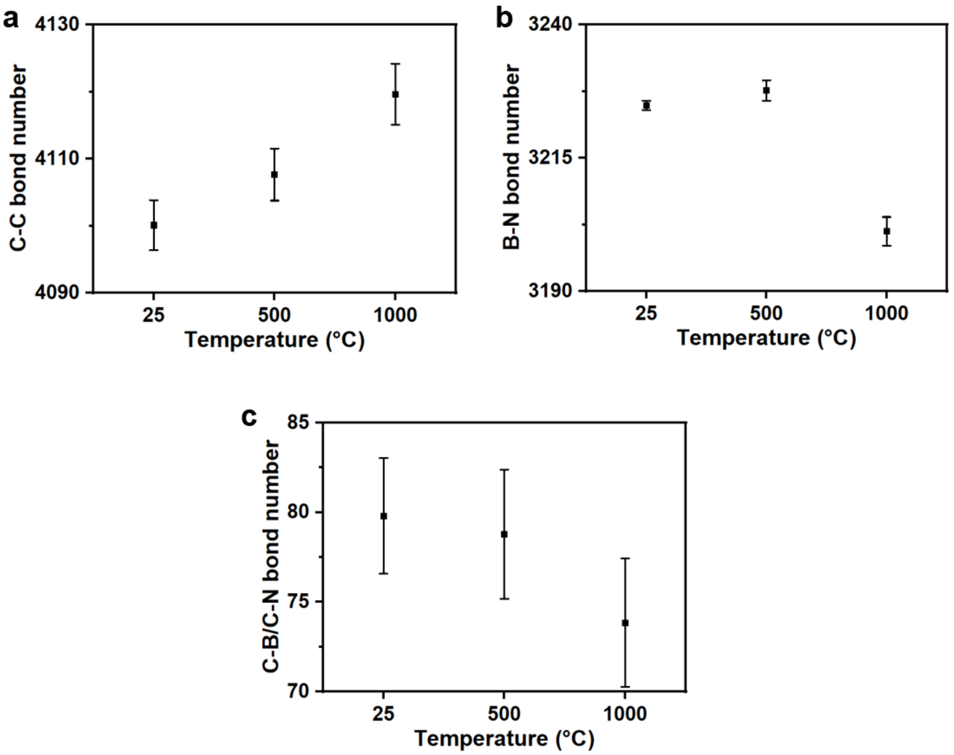

Figure S10. Temperature dependencies of number of (a) C-C bond, (b) B-N bond, and (c) C-B/C-N bond in the system of a-BNGA.

As can be seen in Fig. S10, all the samples remain stable after 1 ns of heat treatment. The bonding of C-C remains stable (Fig. S10a), while the number of covalent bonds between B-N, C-B, and C-N would slightly decrease (less than 7.5%) with the increase of temperature (Fig. S10b and c), which indicate the outstanding thermal stability of a-BNGA and the interface between the a-BN and graphene.

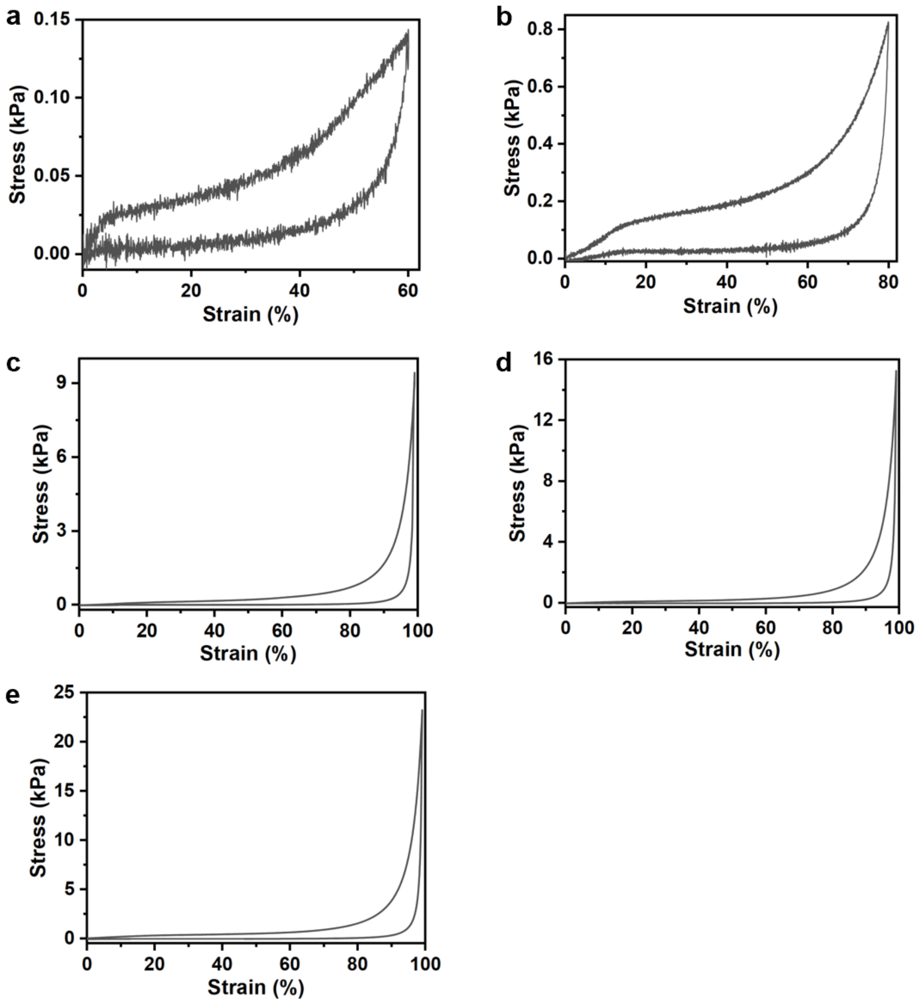

Figure S11. Stress-strain curves of a-BNGA under high recoverable compression strain with different densities. (a-e) 0.4 mg cm^{-3}, 0.6 mg cm^{-3}, 0.8 mg cm^{-3}, 1.1 mg cm^{-3}, 1.3 mg cm^{-3}.

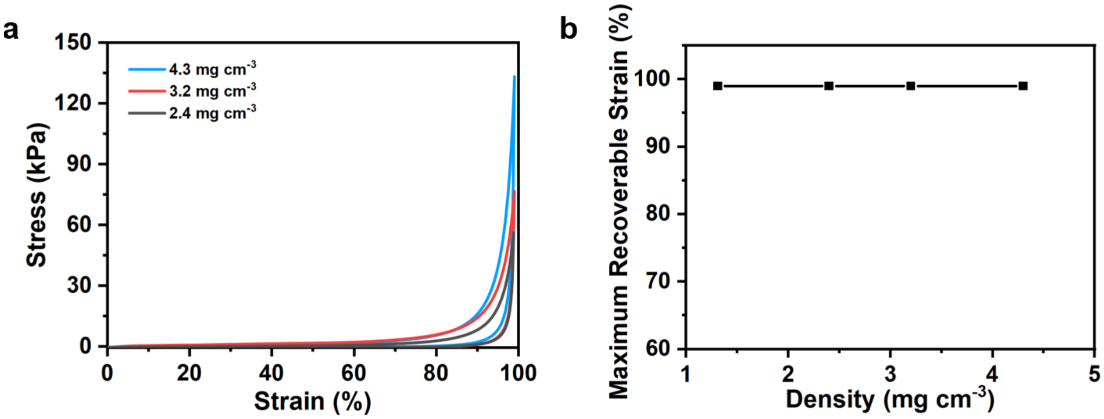

Figure S12. Stress-strain curves of a-BNGA under 99% compression strain with higher densities. Using GO solution with concentration of 4, 6 and 8 mg ml^{-1}, the final density of a-BNGA is 2.4, 3.2 and 4.3 mg cm^{-3}, respectively.

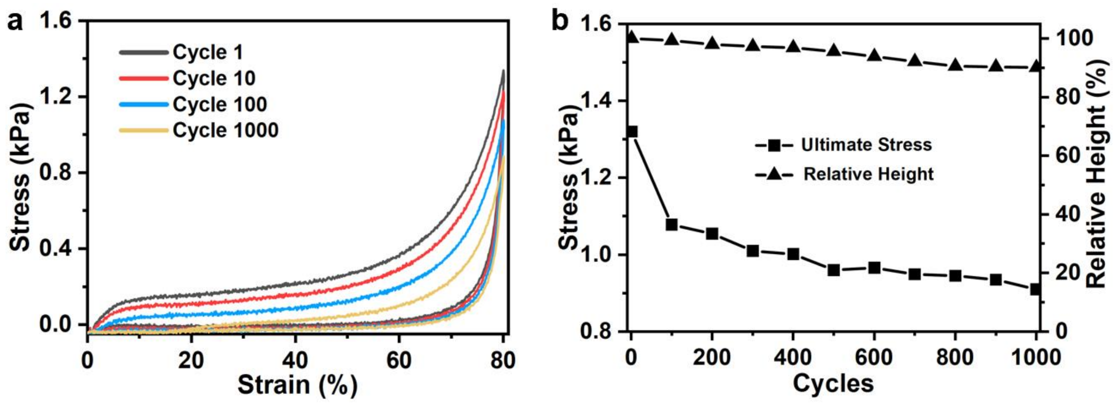

Figure S13. Mechanical durability of typical superelastic a-BNGA. (a) Stress-strain curves of a-BNGA under 80% compression strain for 1,000 cycles. (b) The ultimate stress and relative height for 1,000 compression cycles.

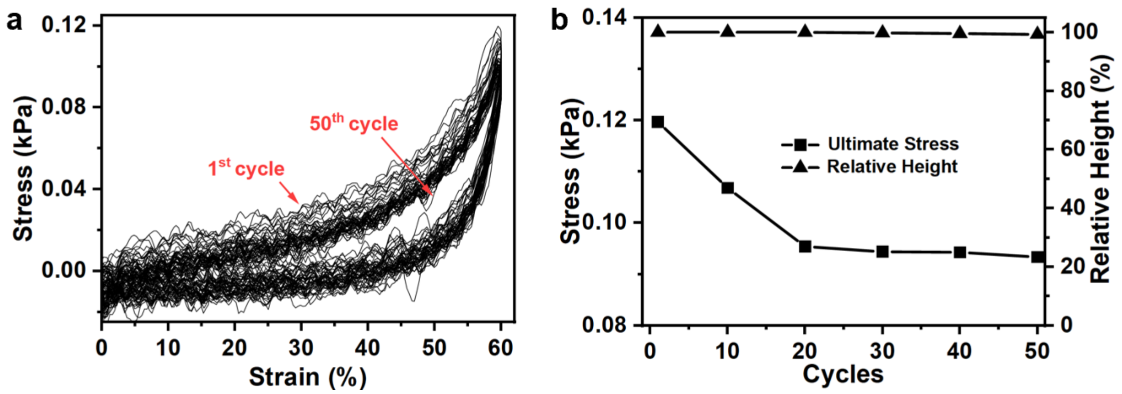

Figure S14. Mechanical durability of a-BNGA with a density of 0.4 mg cm^{-3}. (a) Stress-strain curves of a-BNGA under 60% compression strain for 50 cycles. (b) The ultimate stress and relative height for 50 compression cycles.

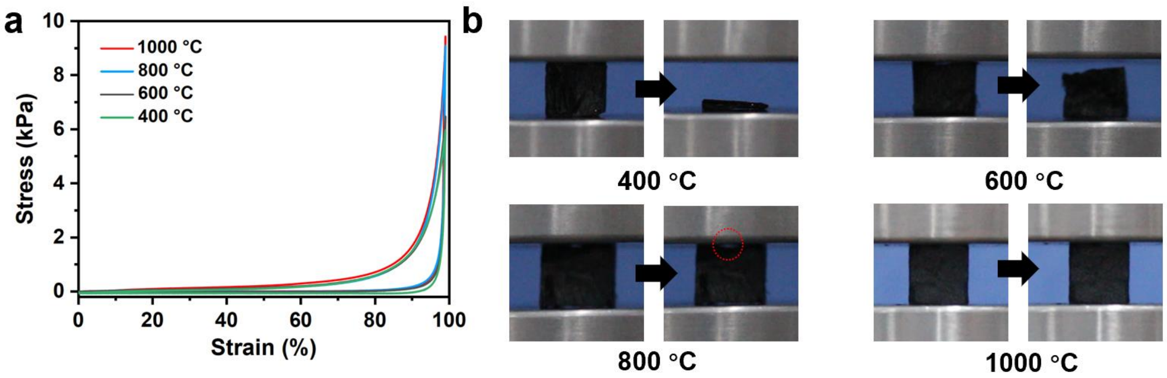

Figure S15. Mechanical properties of a-BNGA (~0.8 mg cm^{-3}) at different annealing temperatures (a) Stress-strain curves of a-BNGA at different annealing temperature. (b) Experimental snapshots of a-BNGA at different annealing temperature after 99% compression strain.

Low annealing temperature leads to incomplete thermal reduction of GO and incomplete dehydrogenation of a-BN, reducing the resilience of the cell wall of a-BNGA.

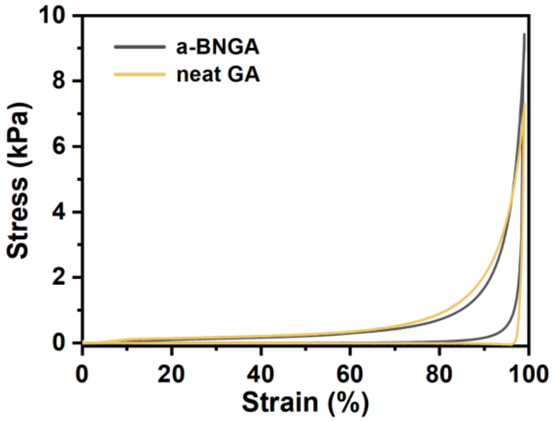

Figure S16. Stress-strain curves of a-BNGA and neat graphene aerogel. The a-BNGA and neat graphene aerogel both have the same graphene volumetric content of ~0.5 mg cm^{-3}.

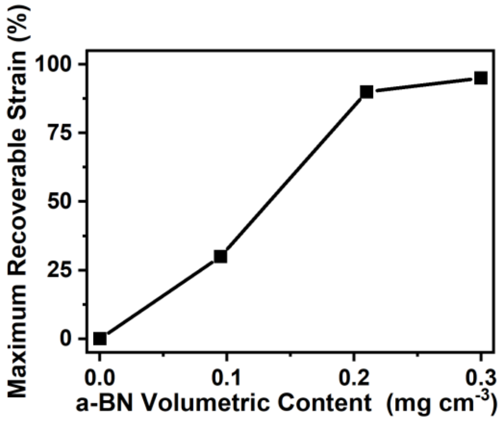

Figure S17. The toughening effect of a-BN deposition on the graphene framework. The maximum elastic deformability of a-BNGA as a function of the a-BN volumetric content. These aerogels have the same graphene volumetric content of ~0.5 mg cm^{-3}. By controlling the input of AB to regulate the a-BN volumetric content.

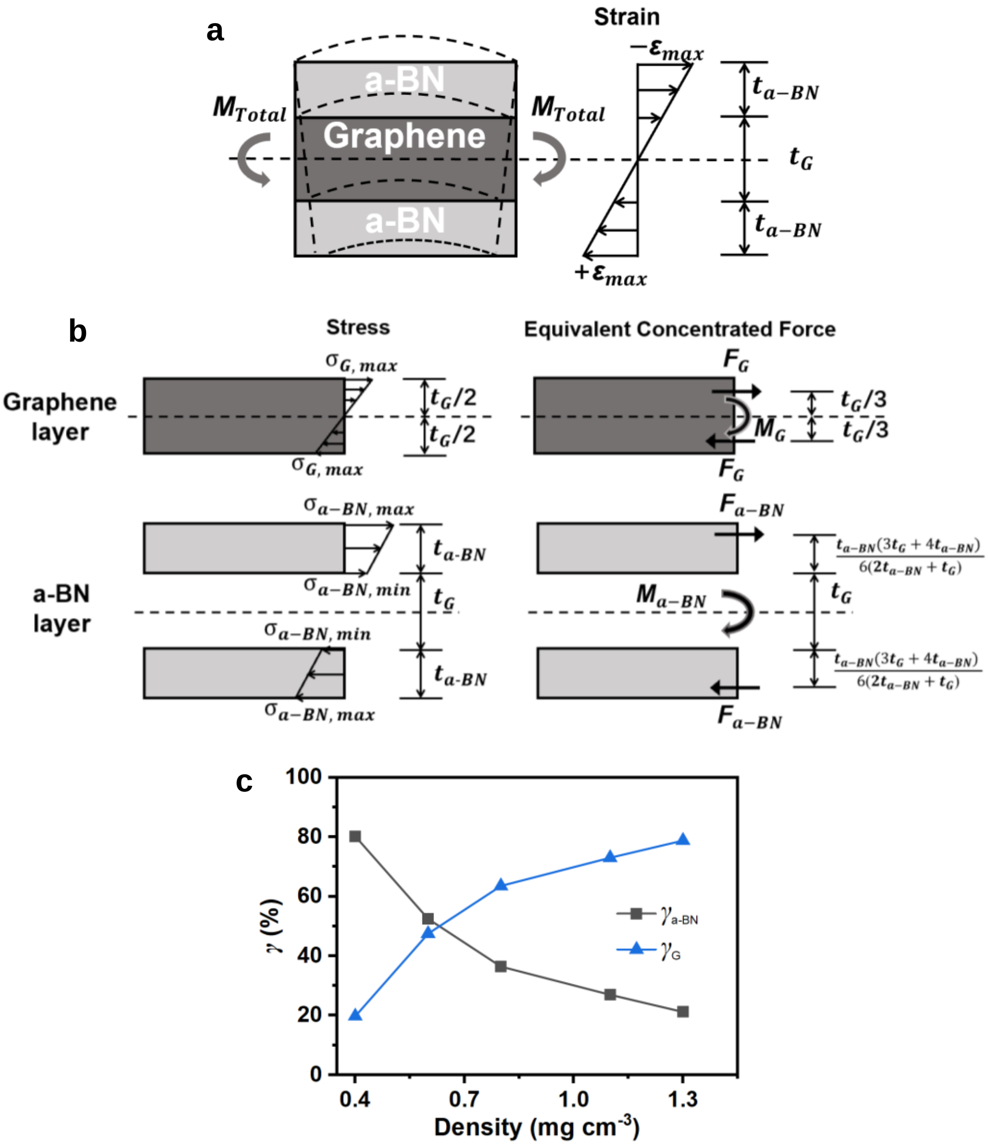

Figure S18. The enhance effect of a-BN nanolayer on the cell walls. (a) Mechanical modeling schematic of a-BNGA with a linearly distributed stress profile along the height in the bending process. (b) The stress and equivalent concentrated force distributions applied on the graphene framework and a-BN nanolayer. (c) γ versus density curves for moment apportion as an index for the a-BN nanolayer effect on the mechanical properties of a-BNGA.

The cell walls of a-BNGA were simplified as a mechanical model of a sandwich slab [19]. According to the classical Kirchhoff hypothesis, requiring that cross-sections remain planar during bending deformation, and the strain distribution along the height of the beam following a linear relationship. The bending moments of the graphene framework and a-BN nanolayer is calculated as:

$$M_G = \frac{E_G \varepsilon_{max} t_G^3}{6(t_G + 2t_{a\text{-}BN})}$$

$$M_{a\text{-}BN}=\frac{E_{a\text{-}BN}\varepsilon_{max}t_{a-BN}(t_G+t_{a-BN})}{(2t_{a-BN}+t_G)}\times[t_G+\frac{t_{a-BN}(3t_G+4t_{a-BN})}{3(2t_{a-BN}+t_G)}]$$

$$\gamma_G=\frac{1}{1+2\frac{E_{a\text{-}BN}}{E_G}\ \beta(1+\ \beta)\left[\beta\frac{(3+4\beta)}{(1+2\beta)}+3\right]}$$

$$\gamma_{a\text{-}BN}=\frac{2\frac{E_{a\text{-}BN}}{E_G}\ \beta(1+\ \beta)\left[\beta\frac{(3+4\beta)}{(1+2\beta)}+3\right]}{1+2\frac{E_{a\text{-}BN}}{E_G}\ \beta(1+\ \beta)\left[\beta\frac{(3+4\beta)}{(1+2\beta)}+3\right]}$$

where E_{G} and $E_{\mathrm{a\text{-}BN}}$ are the Young's modulus of the graphene and a-BN nanolayer (GPa), respectively; t_{G} and $t_{\mathrm{a\text{-}BN}}$ are the thickness of the graphene and a-BN nanolayer at single sides, respectively (nm); M_{G} and $M_{\mathrm{a\text{-}BN}}$ are the bending moments of the graphene and the a-BN undertaken; γ_{G} and $\gamma_{\mathrm{a\text{-}BN}}$ are the partitioned coefficients of bending moments, the values are the ratios of M_{G} and $M_{\mathrm{a\text{-}BN}}$ to M_{Total}, respectively; β is defined as the ratio of $t_{\mathrm{a\text{-}BN}}$ to t_{G}. Due to the effects of reduction defects and layer number of graphene, the value of E_{G} is estimated to 500 Gpa [20,21]. The $E_{\mathrm{a\text{-}BN}}$ is calculated to 55.17 Gpa by MD simulation. The a-BN nanolayer contributes more than 30% of the total moments to enhance a-BNGA when the density below 1 mg cm^{-3}.

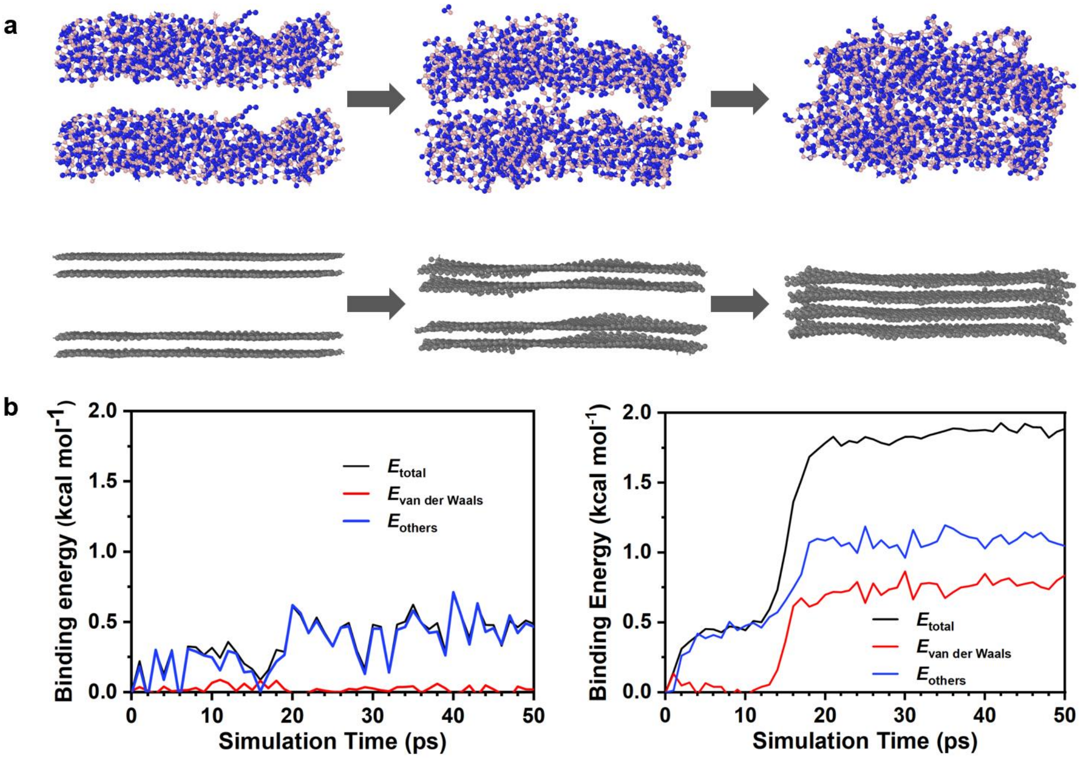

Figure S19. MD simulations of the energy contributed by van der Waals forces. (a) Snapshots of binding process of a-BN to a-BN (upper) and graphene to graphene (lower). (b) Total binding energy and the binding energy contributed by different interatomic interactions of a-BN (left) and graphene (right).

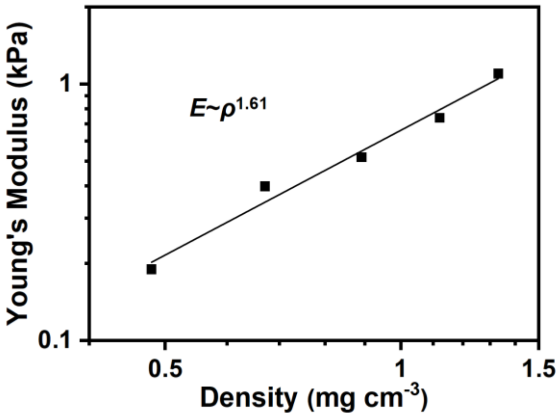

Figure S20. Young’s modulus of a-BNGA at different densities.

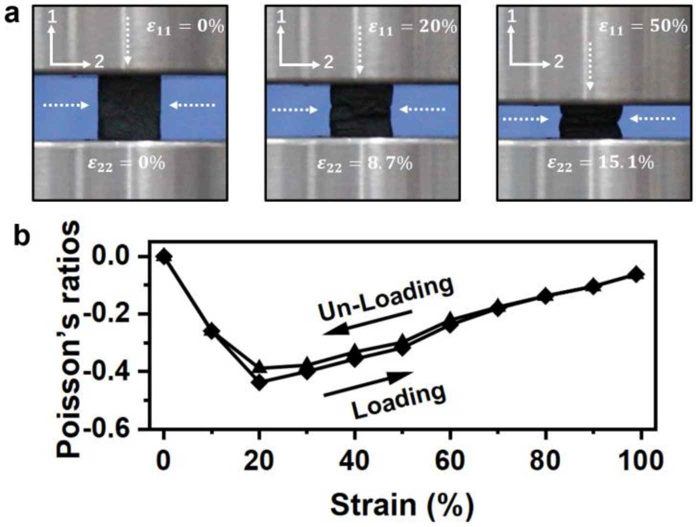

Figure S21. Negative Poisson's ratio behavior of a-BNGA. (a) Experimental snapshots of cross-sectional views of the NPR behavior of a-BNGA (~0.8 mg cm^{-3}) under uniaxial compression. (b) Evolution of the Poisson's ratios ($-\varepsilon_{22}/\varepsilon_{11}$) as a function of compressive strain in one loading un-loading cycle.

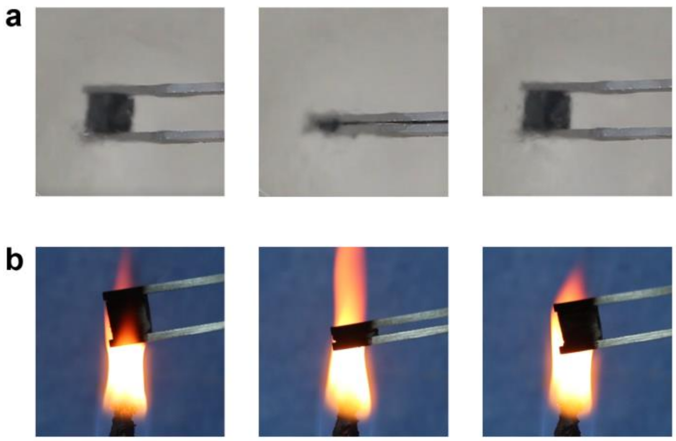

Figure S22. Measurement of superelasticity of a-BNGA over a wide temperature. (a) In liquid N_2 (-196 ℃). (b) In flame (~500 ℃).

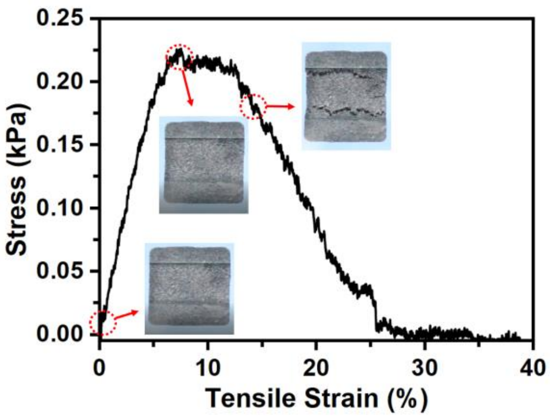

Figure S23. Experimental snapshots and stress-strain curve of a-BNGA under tensile loading. The a-BNGA exhibits high tensile strain (7.5%) and ductile fracture behavior.

The tensile property was studied using samples with a length of 20 mm by an Instron 3365 at a loading rate of 10 mm min^{-1}. The lower end and upper end were tightly fixed. During the test, it can be observed that there was no slip and deformation at the upper and lower ends, ensuring the accuracy of tensile test.

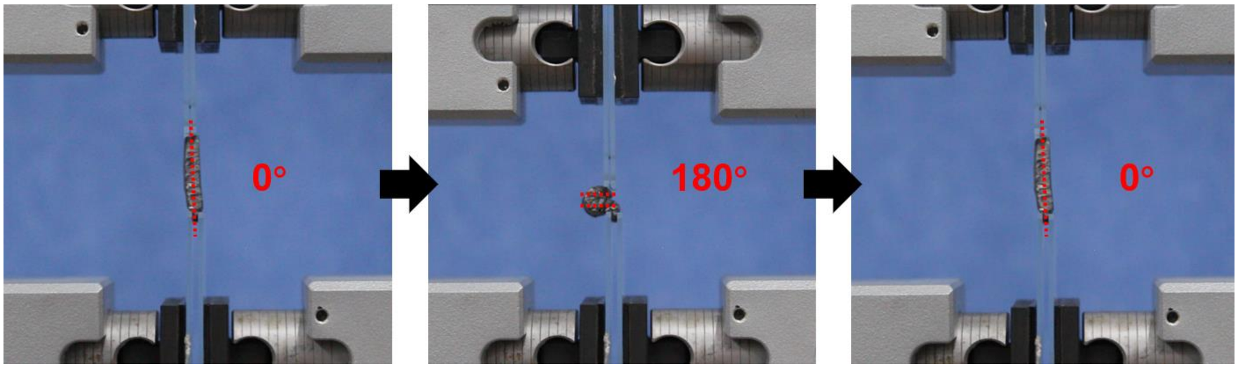

Figure S24. Recoverable angle of a-BNGA under two-point buckling.

The bendability was studied using samples with a length of 20 mm and thickness of 3.5 mm by an Instron 3365 at a loading rate of 10 mm min^{-1}. During the test, the lower end was fixed, and the upper end only contacted the fixture. Therefore, the recovery process after bending was the self-recovery of the sample.

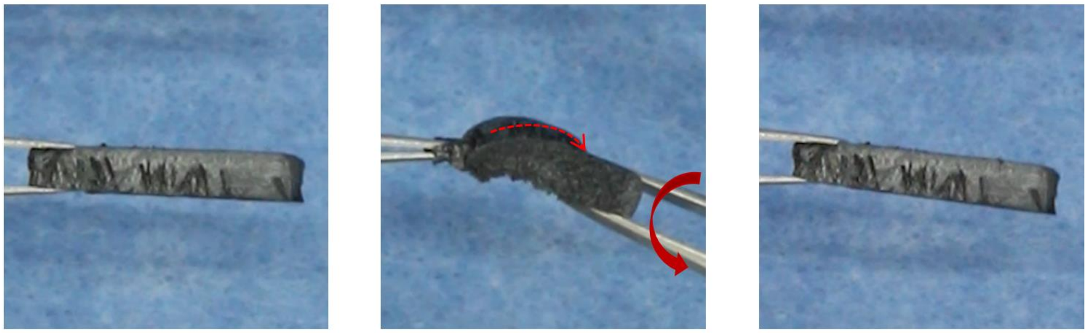

Figure S25. Experimental snapshots of one torsion cycle of a-BNGA. The a-BNGA exhibits a torsional angle up to 90 °with little morphology change.

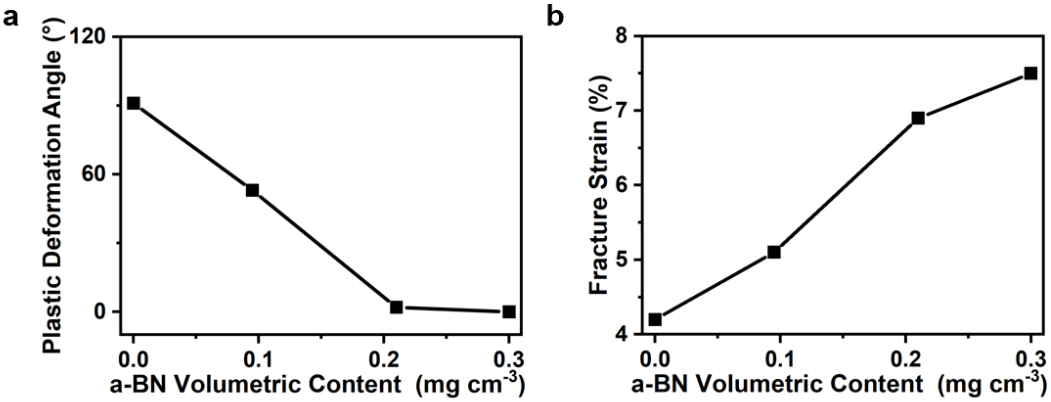

Figure S26. Effect of a-BN deposition on bending and tensile properties. (a) The plastic deformation angle after two-point buckling (bending angle 180 °) and (b) fracture strain of a-BNGA as a function of the a-BN volumetric content. These aerogels have the same graphene volumetric content of ~0.5 mg cm^{-3}. By controlling the input of AB to regulate the a-BN volumetric content.

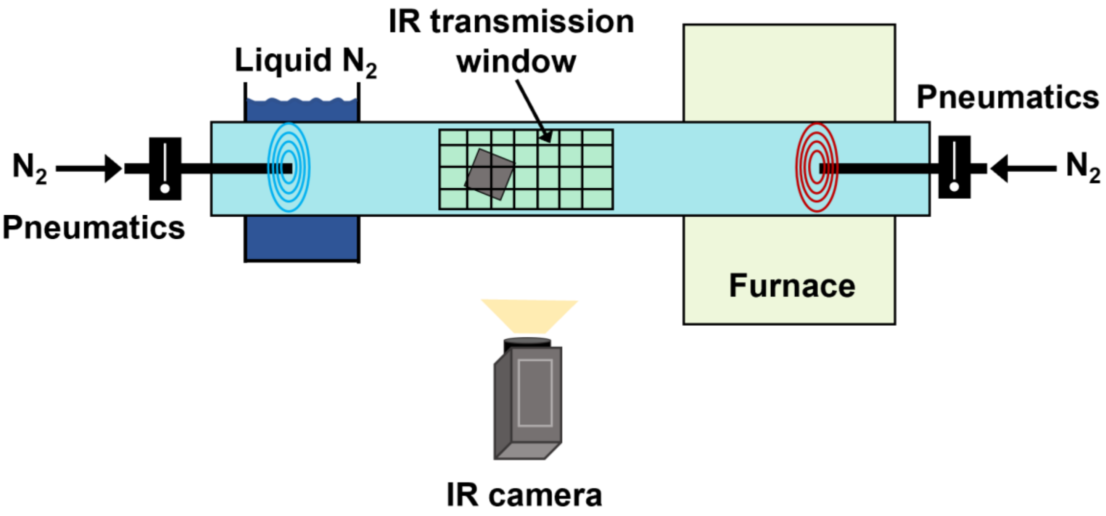

Figure S27. A thermal shock testing system for lightweight materials. Two copper pistons were fixed at both ends of the quartz tube. The right end of quartz tube passed through the furnace and the left end passed container containing liquid nitrogen. The left piston was cooled by liquid N_2 as cold source and the right one was heated by furnace as hot source. The aerogels were moved between the cold and hot sources by N_2 gas of the pneumatic devices. One IR transmission window was opened at the middle of the tube for IR measurement. By controlling the gas flow of the pneumatics devices, the aerogels can be heated or cooled easily at a specific rate to realize the thermal shock test.

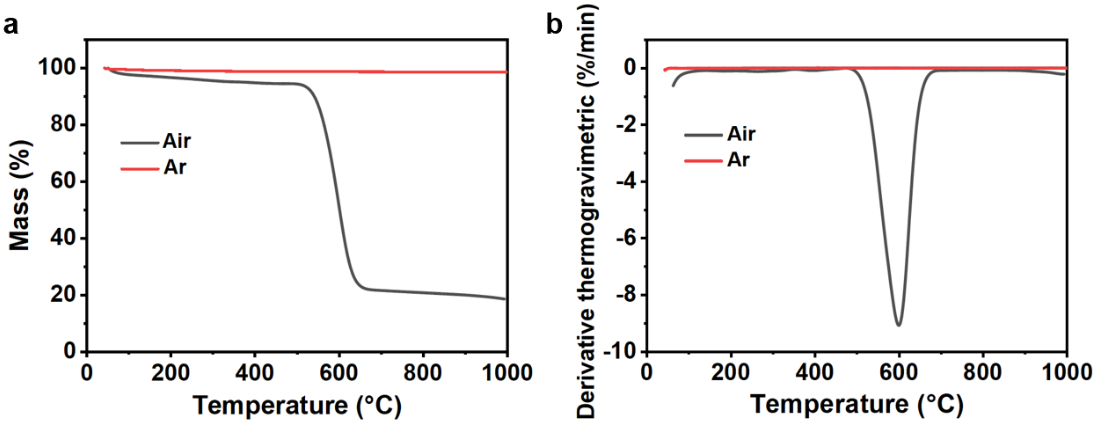

Figure S28. The thermal stability of a-BNGA at ambient pressure and high temperature in air and Ar. (a) The thermogravimetric (TG) analysis and (b) derivative thermogravimetric (DTG) analysis of a-BNGA.

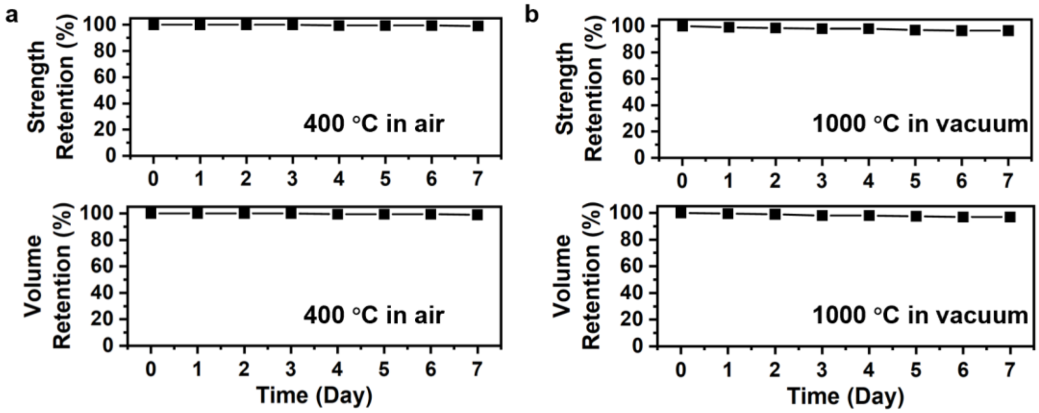

Figure S29. Strength and volume variation of a-BNGA in long-term high temperature condition. (a) 400 ℃ in air, (b) 1,000 ℃ in vacuum.

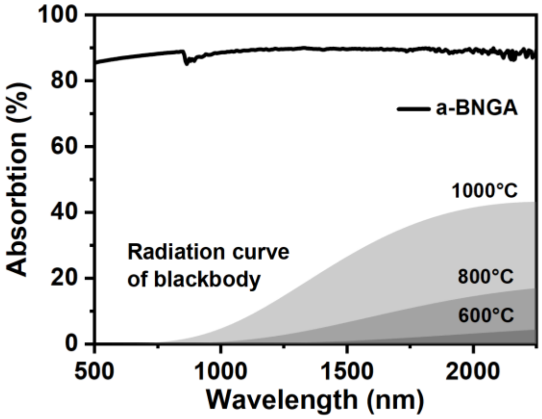

Figure S30. UV-vis-NIR reflection spectra of a-BNGA. Radiation curves of blackbody at 600, 800 and 1,000 ℃ is indicated by shade region respectively.

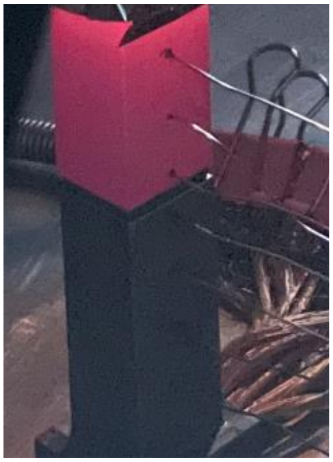

Figure S31. Reference bar setup optical photograph in high temperature test in vacuum. At this time, the temperature of the upper reference bar is above 525 ℃, while the temperature of the lower reference bar is below 40 ℃.

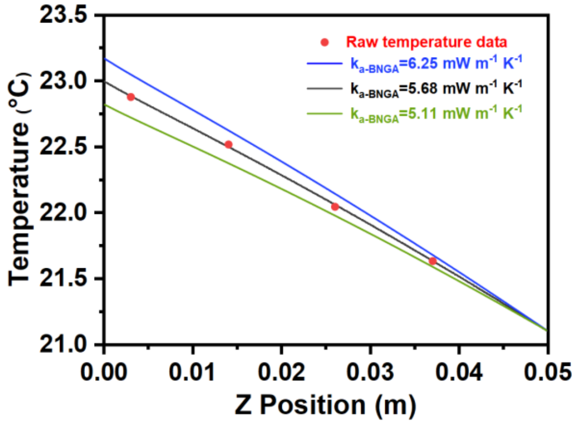

Figure S32. The nonlinear fitting curves of raw temperature data in vacuum. When the result of thermal conductivity fluctuates by 10%, the three fitting curves have obvious differentiation, indicating great sensitivity of the test. The fitting curve is close to a linear curve (almost straight in the figure), but it is still strictly nonlinear. The reason for the good linearity is that we try to make enough cooling at the bottom of the lower reference bar in this test, so that one-dimensional thermal conduction is still dominant.

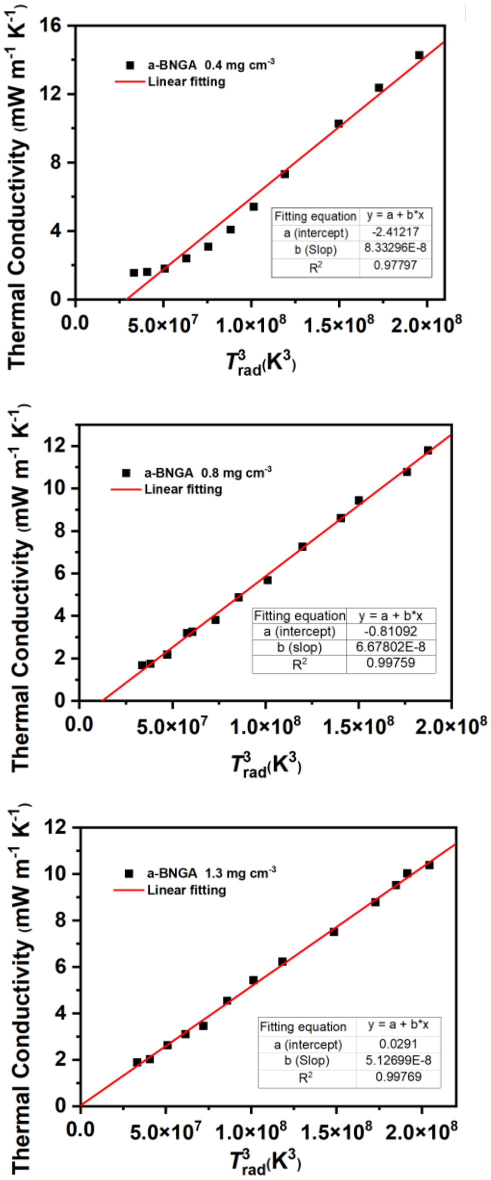

Figure S33. Replot of linear fitting performed according to T_{rad}^3 and corresponding total thermal conductivity.

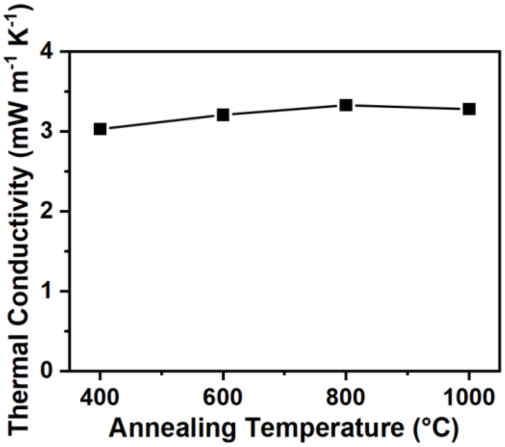

Figure S34. Thermal conductivity of a-BNGA (~0.8 mg cm^{-3}) in vacuum at 210℃ with different annealing temperatures. There is almost no change in vacuum thermal conductivity when the annealing temperature is higher than 600 ℃ (~ 2%). When the annealing temperature is further reduced to 400 ℃, the thermal conductivity decreases slightly (~ 7.5%) due to the incomplete thermal reduction of GO and incomplete dehydrogenation of a-BN enhancing phonon scattering.

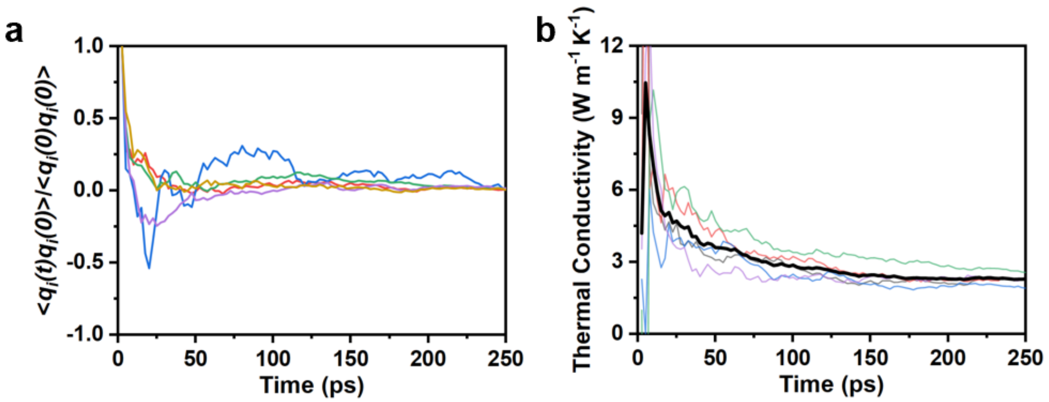

Figure S35. Thermal conductivity of a-BN. (a) The normalized HCACFs as a function of simulation time for 5 individual EMD simulations. (b) Thermal conductivity of 5 individual EMD simulations (colored lines) and the average thermal conductivity (black line).

The average thermal conductivity of a-BN sample is 2.31 W m^{-1} K^{-1}, which is significantly lower than the thermal conductivity of graphene and hBN.

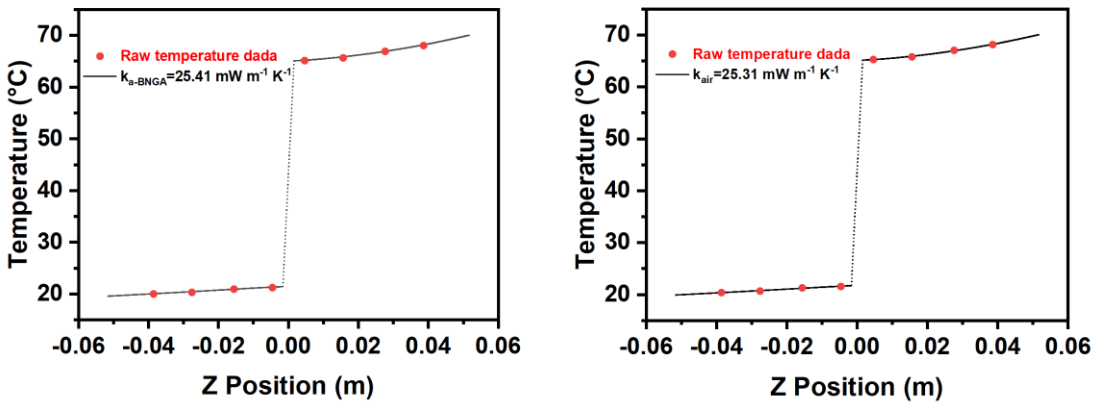

Figure S36. The nonlinear fitting curves of raw temperature data in atmosphere. Nonlinearity of curves shows that heat flow passes from the top of the upper bar to the bottom of the lower bar through the middle sample, meanwhile accompanied by thermal convection and radiation with the surrounding environment obviously.

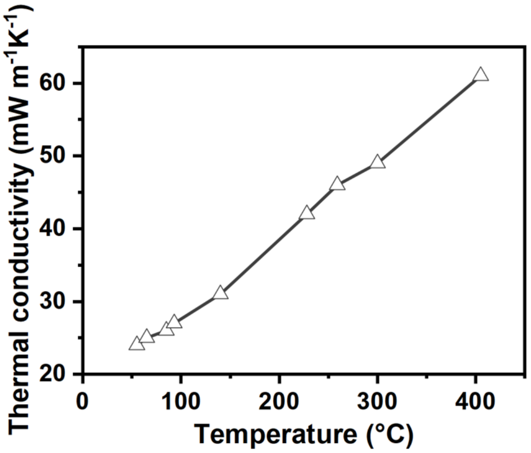

Figure S37. Thermal conductivity of a-BNGA (~0.8 mg cm^{-3}) in air atmosphere as a function of temperature.

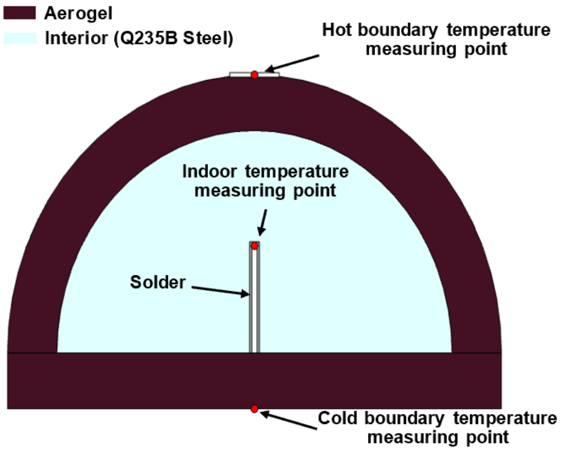

Figure S38. The internal structure of the lunar base model. The interior is a hemisphere solid with a diameter of 40 mm. There is a straight hole with a depth of 10 mm and a diameter of 1 mm in the center for placing thermocouples with a diameter of 0.5 mm. In order to minimize the measurement error caused by the thermal contact resistance, indium-solder is filled as a thermal interface material in the gap between the thermocouple and the hole. All the surfaces of the hemisphere are covered with an aerogel of 5 mm thickness.

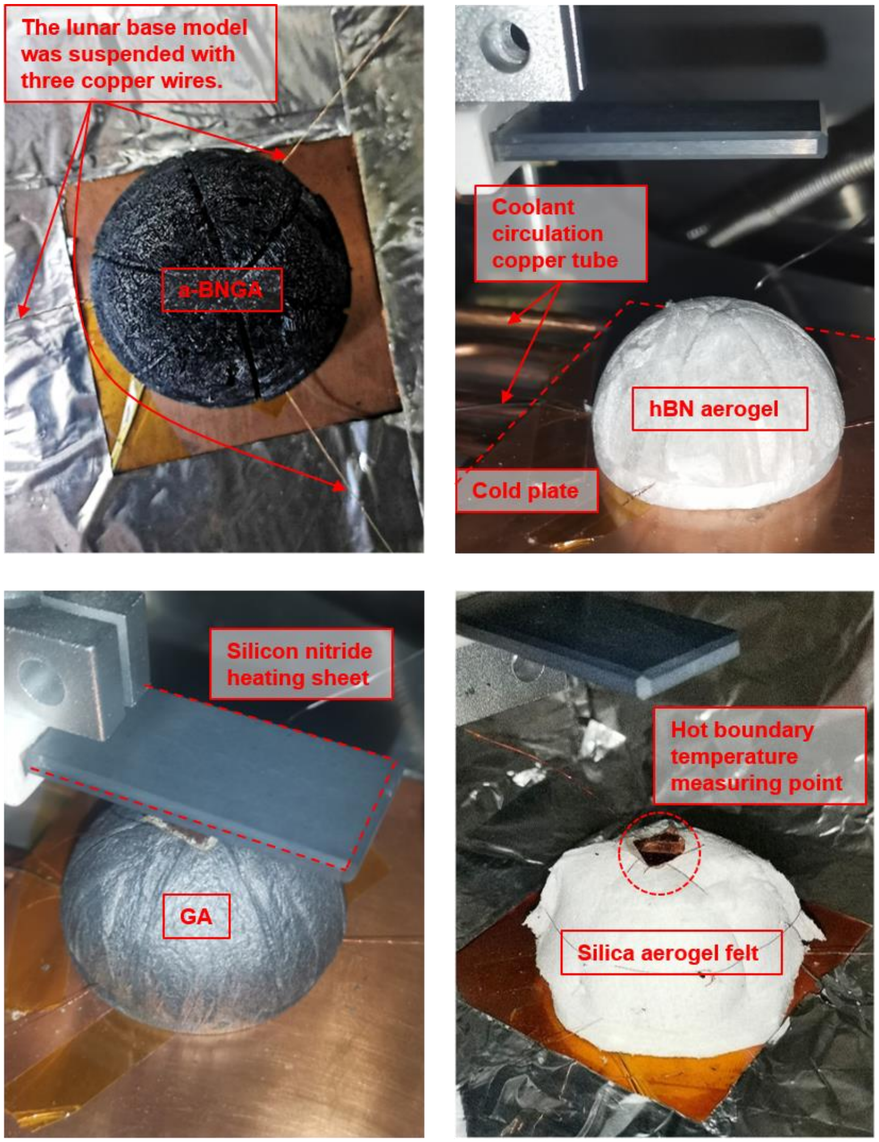

Figure S39. Optical photographs of lunar base model. The interior steel covered by a-BNGA, hBN aerogel, GA and silica aerogel felt, respectively. In this demonstration, the a-BNGA, hBN aerogel and GA all with ultralow density (< 4 mg cm^{-3}). Due to the poor mechanical properties of commercial silica aerogel felt, it cannot be directly made into a standard spherical surface. In order to fully demonstrate the thermal insulation performance of commercial silica aerogel felt, the thickness of commercial silica aerogel felt was guaranteed to be at least 5 mm at any geometric position.

	T_1[℃]	T_2[℃]	**T_3[℃]**	T_4[℃]	T_5[℃]	T_6[℃]	T_7[℃]	**$\kappa_{a\text{-}BNGA}$ [mW m^{-1} K^{-1}]**
**0.4 [mg cm^{-3}]**	79.727	79.441	**79.382**	15.384	15.341	15.312	15.270	**1.57**
	121.564	121.120	**120.911**	15.899	15.855	15.814	15.738	**1.63**
	168.713	167.859	**167.408**	16.608	16.550	16.460	16.383	**1.82**
	218.015	216.245	**215.840**	17.286	17.186	17.047	16.921	**2.40**
	261.735	258.157	**257.614**	18.886	18.721	18.514	18.348	**3.09**
	303.937	297.587	**295.078**	20.840	20.582	20.283	20.034	**4.09**
	346.308	333.725	**329.238**	23.313	22.906	22.478	22.128	**5.43**
	400.567	374.644	**371.462**	25.655	25.042	24.378	23.850	**7.32**
	476.115	444.788	**433.099**	31.897	30.897	29.818	28.943	**10.27**
	526.273	489.851	**472.267**	38.594	37.405	35.965	34.748	**12.36**
	567.738	529.371	**506.530**	47.257	45.910	44.055	42.589	**14.27**
**0.8 [mg cm^{-3}]**	81.700	81.360	**81.343**	16.541	16.507	16.481	16.439	**1.69**
	107.179	106.636	**106.503**	16.744	16.712	16.680	16.607	**1.76**
	152.882	151.898	**151.479**	16.511	16.471	16.395	16.275	**2.19**
	199.233	197.822	**197.305**	16.006	15.902	15.734	15.558	**3.20**
	210.814	208.729	**208.245**	16.016	15.896	15.734	15.561	**3.27**
	254.108	251.681	**250.683**	17.623	17.420	17.193	16.962	**3.82**
	292.326	290.915	**288.104**	19.624	19.329	18.992	18.671	**4.88**
	338.720	332.262	**329.021**	22.880	22.515	22.048	21.639	**5.68**
	387.682	377.316	**371.851**	27.869	27.309	26.646	26.102	**7.27**
	434.623	420.164	**414.422**	32.399	31.700	30.800	30.086	**8.61**
	454.302	440.840	**432.136**	35.155	34.289	33.301	32.505	**9.44**

	506.148	490.650	**478.649**	38.361	37.330	36.035	34.973	**10.78**
	531.876	516.796	**503.791**	40.829	39.662	38.179	36.983	**11.63**
	79.244	79.151	**79.073**	15.998	15.964	15.934	15.873	**1.88**
	119.845	119.553	**119.343**	16.845	16.774	16.737	16.660	**2.02**
	169.682	168.786	**168.175**	16.733	16.655	16.548	16.422	**2.62**
	211.457	210.713	**209.947**	17.231	17.130	16.952	16.790	**3.11**
	247.591	246.820	**245.982**	18.964	18.779	18.588	18.385	**3.46**
	291.097	289.345	**288.283**	20.812	20.574	20.250	19.948	**4.54**
**1.3 [mg cm^{-3}]**	335.058	333.127	**329.663**	22.499	22.142	21.708	21.310	**5.42**
	375.097	373.040	**370.796**	23.980	23.521	22.933	22.443	**6.23**
	437.480	433.886	**432.359**	28.995	28.341	27.527	26.834	**7.51**
	476.283	475.002	**474.859**	34.720	33.916	32.860	31.973	**8.78**
	496.043	495.915	**494.774**	36.939	35.943	34.794	33.835	**9.52**
	507.863	506.718	**505.181**	37.914	37.039	35.786	34.726	**10.03**
	531.051	529.988	**525.752**	39.170	38.081	36.772	35.639	**10.39**

Table S1. Raw temperature data and results for the total thermal conductivity of a-BNGA test in vacuum. T_3 is the approximate hot side temperature here.

Movie S1.
Mechanical compression tests of a-BNGA.
Movie S2.
Fatigue test of a-BNGA.
Movie S3.
Mechanical tensile, bending, torsion and eccentric compression tests of a-BNGA.
Movie S4.
Flexible deformability test of a-BNGA.
Movie S5.
Thermal shock test of a-BNGA

Supplementary References

1. van Duin ACT, Dasgupta S and Lorant F *et al.* ReaxFF: A reactive force field for hydrocarbons. *J Phys Chem A* 2001; **105**: 9396-409.
2. van Duin ACT, Strachan A and Stewman S *et al.* ReaxFFSiO reactive force field for silicon and silicon oxide systems. *J Phys Chem A* 2003; **107**: 3803-11.
3. Pai SJ, Yeo BC and Han SS. Development of the ReaxFFCBN reactive force field for the improved design of liquid CBN hydrogen storage materials. *Phys Chem Chem Phys* 2016;**18**:1818-27.
4. Liu Y, Sun Q and Yu P *et al.* Effects of high and low salt concentrations in electrolytes at lithium–metal anode surfaces using DFT-ReaxFF hybrid molecular dynamics method. *J Phys Chem Lett* 2021; **12**: 2922-9.
5. Berendsen HJC, Postma JPM and van Gunsteren WF *et al.* Molecular dynamics with coupling to an external bath. *J Chem Phys* 1984; **81**: 3684-90.
6. Plimpton S. Fast parallel algorithms for short-range molecular dynamics. *J Comput Phys* 1995; **117**: 1-19.
7. Liu Y, Pan Y and Yin D *et al.* Large-scale generation and characterization of amorphous boron nitride and its mechanical properties in atomistic simulations. *J Non-Cryst Solids* 2021; **558**: 120664.
8. Stone AJ and Wales DJ. Theoretical studies of icosahedral C60 and some related species. *Chem Phys Lett* 1986; **128**: 501-3.
9. Allen MP and Tildesley DJ. *Computer simulation of liquids*. Oxford University Press, 2017.
10. Hill R. The elastic behaviour of a crystalline aggregate. *Proc Phys Soc A* 1952; **65**: 349-54.
11. Kınacı A, Haskins JB and Sevik C *et al.* Thermal conductivity of BN-C nanostructures. *Phys Rev B* 2012; **86**: 115410.
12. Schelling PK, Phillpot SR and Keblinski P. Comparison of atomic-level simulation methods for computing thermal conductivity. *Phys Rev B* 2002; **65**: 144306.
13. Hanson K. ASTM D 5470 TIM material testing. In: *20th Annual IEEE semiconductor thermal measurement and management symposium,* Dallas, TX, USA. Piscataway, NJ: IEEE Press, 2006, 50–3.
14. AK Steel 304/304L Stainless Steel product data bulletin.
15. Lee D, Stevens PC and Zeng SQ *et al.* Thermal characterization of carbon-opacified silica aerogels. *J Non-Cryst Solids* 1995; **186**: 285-90.
16. Q235 Steel-Punchlist Zero-properties, equivalent grades, and applications.
17. Paige DA, Foote MC and Greenhagen BT *et al.* The lunar reconnaissance orbiter diviner lunar radiometer experiment. *Space Sci Rev* 2010; **150**: 125-60.
18. Mamoru O and Akira K. Lumped-parameter modeling of heat transfer enhanced by sinusoidal motion of fluid. *Int J Heat Mass Tran* 1991; **34**: 3083-95.

19. Zhang Q, Lin D and Deng B *et al*. Flyweight, superelastic, electrically conductive, and flame‐retardant 3D multi‐nanolayer graphene/ceramic metamaterial. *Adv Mater* 2017; **29**: 1605506.
20. Jing N, Xue Q and Ling C *et al*. Effect of defects on Young's modulus of graphene sheets: a molecular dynamics simulation. *RSC Adv* 2012; **2**: 9124.
21. Zhong T, Li J and Zhang K. A molecular dynamics study of Young's modulus of multilayer graphene. *J Appl Phys* 2019; **125**: 175110.
